# Does Heat Play a Role in the Observed Behavior of Aqueous Photobatteries?

**DOI:** 10.1021/acsenergylett.3c01627

**Published:** 2023-10-12

**Authors:** Arvind Pujari, Byung-Man Kim, Farheen N. Sayed, Kate Sanders, Wesley M. Dose, Angus Mathieson, Clare P. Grey, Neil C. Greenham, Michael De Volder

**Affiliations:** † Cavendish Laboratory, Department of Physics, University of Cambridge, Cambridge CB3 0HE, U.K.; ‡ Institute for Manufacturing, Department of Engineering, University of Cambridge, Cambridge CB3 0FE, U.K.; § Department of Chemistry, University of Cambridge, Cambridge CB2 1EW, U.K.; ∥ School of Chemistry, University of New South Wales, Sydney, NSW 2052, Australia

## Abstract

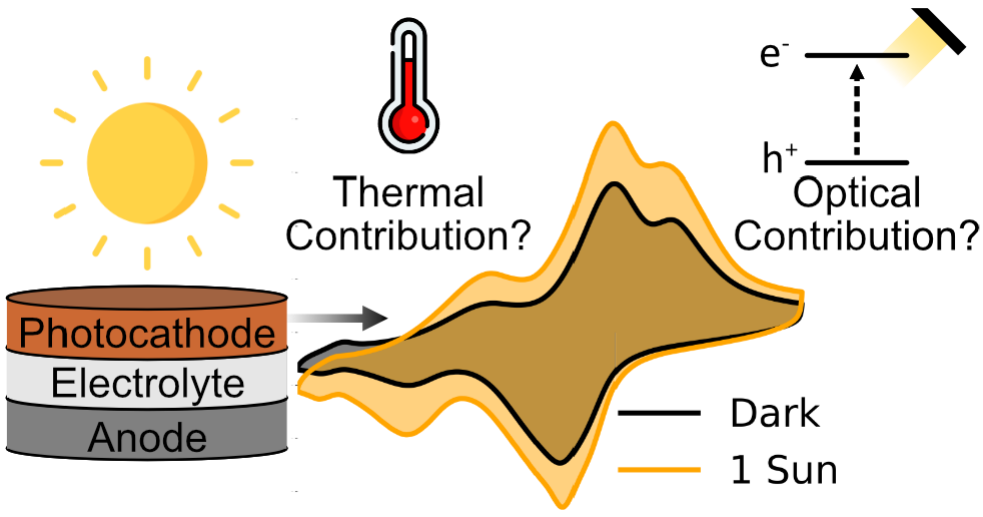

Light-rechargeable photobatteries have emerged as an elegant solution to address the intermittency of solar irradiation by harvesting and storing solar energy directly through a battery electrode. Recently, a number of compact two-electrode photobatteries have been proposed, showing increases in capacity and open-circuit voltage upon illumination. Here, we analyze the thermal contributions to this increase in capacity under galvanostatic and photocharging conditions in two promising photoactive cathode materials, V_2_O_5_ and LiMn_2_O_4_. We propose an improved cell and experimental design and perform temperature-controlled photoelectrochemical measurements using these materials as photocathodes. We show that the photoenhanced capacities of these materials under 1 sun irradiation can be attributed mostly to thermal effects. Using *operando* reflection spectroscopy, we show that the spectral behavior of the photocathode changes as a function of the state of charge, resulting in changing optical absorption properties. Through this technique, we show that the band gap of V_2_O_5_ vanishes after continued zinc ion intercalation, making it unsuitable as a photocathode beyond a certain discharge voltage. These results and experimental techniques will enable the rational selection and testing of materials for next-generation photo-rechargeable systems.

Solar energy is an attractive replacement for fossil fuels.^1^ However, the intermittent nature of solar irradiation means that energy storage devices are needed to address the mismatch between solar energy demand and supply.^2^ At a smaller scale, the ability to combine light energy harvesting and storage is useful for powering off-grid sensors and other portable devices as a part of the Internet of Things (IoT) revolution.^3^ This dual functionality must be achieved within an extremely small footprint to minimize the size and increase the energy density of these devices.

Light-rechargeable photobatteries have emerged as an elegant solution to this problem. Such devices were first conceived in the 1970s, when Tributsch^4^ reported layered transition metal dichalcogenides as a promising electrode for the photo-intercalation of copper and hydrogen ions. In recent years, there has been a revival in the studies of light–battery interactions in two-electrode systems. These include the light-assisted delithiation of a dye-sensitized LiFePO_4_ cathode^5^ and the photocharging of a halide perovskite.^6^ Subsequently, a series of papers on the photocharging and photoenhanced behaviors of zinc-ion and lithium-ion batteries were reported, using VO_2_ and V_2_O_5_.^7−13^ Other materials reported as photocathodes include lead halide perovskites coupled with a Te cathode,^11^ lead-free perovskites,^14^ MoS_2_,^15^ TiO_2_,^16,17^ LiMn_2_O_4_,^18−20^ SnO_2_,^21^ organic porous cages,^22,23^ and MoO_3_.^24^

However, very few papers reporting photocathode materials consider the relative band positions of the photocathode and the cathode de-intercalation and anode plating potentials. Figure 1(a) shows the relationship between the band positions of a semiconducting photocathode and its ability to photocharge. Upon illumination, the populations of electrons and holes are described by the conduction band quasi-Fermi potential (*E*_Fc_) and the valence band quasi-Fermi potential (*E*_Fv_), respectively. For bias-free photocharging to be possible, the conduction band quasi-Fermi potential (*E*_Fc_) must be higher than the anode plating potential of the battery, as shown for Cathode Material 1 in Figure 1(a). This enables the transfer of electrons to the anode to participate in zinc reduction at the anode. Simultaneously, the valence band quasi-Fermi potential (*E*_Fv_) of the photocathode must be lower than the de-intercalation potential of the cathode to enable holes to participate in zinc ion de-insertion. Should either of these conditions be unfulfilled (as shown for Cathode Material 2 in Figure 1(a)), an external bias will be needed to ensure that the photogenerated electrons and holes have enough energy to participate in anodic and cathodic processes, respectively; this is commonly referred to as photoassisted charging. Further considerations needed for band alignments in photobatteries, such as the measurement of conduction band positions, can be found in Supplementary Note 1.

**Figure 1:**
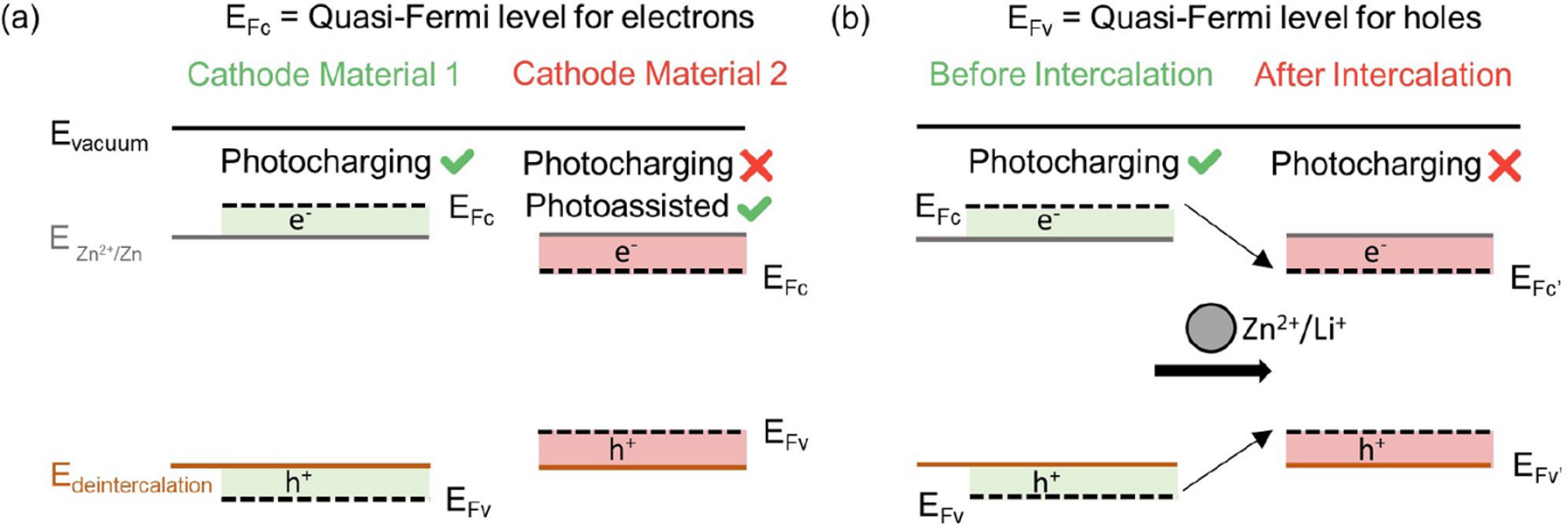
Optical considerations while conducting experiments on light-rechargeable photobatteries. (a) Band structures of two hypothetical cathode materials. When the photocathode is illuminated, the populations of electrons and holes are represented by their respective quasi-Fermi levels. Depending on the relative position of these levels and the anode/cathode plating and de-intercalation potentials, photocharging may (Cathode Material 1) or may not (Cathode Material 2) be possible. (b) Schematic highlighting that intercalation can shift the positions of the electron and hole quasi-Fermi levels, affecting the material’s ability to be photocharged.

Another complication arises from the fact that the band gap and band structure of most semiconductors shift upon intercalation,^10,25−28^ resulting in changed quasi-Fermi levels for electrons and holes, as shown in Figure 1(b). This could result in photocharging not being able to proceed after a certain number of ions are de-intercalated, restricting the photocapacity of the system. The band gap could be blue-shifted or red-shifted due to Burstein–Moss shifts from added electrons^29^ or lost entirely should the number of ions inserted exceed the degenerate limit of the semiconductor. Hence, *operando* tracking of the optical spectra of photocathodes as a function of their state of charge is essential to capture these effects.

Additionally, thermal contributions from illumination to photoenhanced behavior are frequently overlooked. The operating principle of these photobatteries relies on the formation of electron–hole pairs to enhance capacities under illumination (by LED emitters or solar simulators), or to charge the cell. This can lead to significant heat build-up in the system (up to 60 °C under 1 sun^30^). It is well known that temperature can improve the kinetics of electrochemical reactions and reduce charging overpotentials,^31,32^ improving battery performance. Therefore, any thermal contributions must be subtracted before enhanced capacities can be attributed exclusively to light-enhancement effects. We elucidate the contributions of illumination to the heating of photobatteries in Supplementary Note 2.

Here, we focus on bifunctional photocathodes which can both absorb light and intercalate ions. We propose an alternative cell design for the photoelectrochemical measurement of zinc-ion photobatteries where the metal anode is placed directly on a temperature-controlled stage to enhance cooling by heat conduction during measurements. Using this design, we demonstrate simple control experiments to decouple the effects of heat and light on two widely studied photoactive cathode materials, V_2_O_5_ and LiMn_2_O_4_, against a zinc anode in an aqueous electrolyte. This cell design can be extended to other air-stable anodes.

We also investigate the ability of V_2_O_5_-based photocathodes to be photocharged by attempting to charge a fully discharged cell solely through illumination. “Photocharging” refers to the flow of electrons from the photocathode to the anode during illumination. One way to perform this experiment correctly is to first fully discharge the cell and then short-circuit it to allow a path for electrons to flow from the photocathode to the anode. Then, when the sample is illuminated, a positive current should be seen. However, many publications^7,10,24,33^ instead refer to the increase in open-circuit voltage (OCV) upon illumination as “photocharging”, although no electrons can flow under these conditions.

Thermal effects can result in a decrease in overpotentials and improved electrochemical performance due to improved ionic mobility in the electrolyte or heating of the cathode.^34^ We demonstrate this decrease in impedance in Figure S1, where we measure the impedance for a V_2_O_5_–Zn cell at 50% state of charge (SOC) (discharged to 0.7 V) at 19 and 32 °C. The value of the charge-transfer resistance (*R*_CT_) decreases from 819.9 Ω at 19 °C to 378.2 Ω at 32 °C, indicating that elevated temperatures under 1 sun illumination can drastically reduce impedance.

Additionally, thermal effects can lead to accelerated activation of a material’s capacity in certain cathodes like V_2_O_5_. The effect of generated heat on the capacity of these batteries is shown in Figure 2(a), where a V_2_O_5_–Zn coin cell is cycled at a current density of 500 mA/g under 1 sun illumination at room temperature (varying between 19 and 26 °C) and in a 34 °C (the maximum cell temperature recorded under these conditions) incubator to mimic the thermal effects of 1 sun irradiation. The cell cycled in the incubator at 34 °C and the cell cycled under 1 sun illumination both show a higher initial capacity than the cell cycled at room temperature, indicating that thermal contributions can manifest in increased capacities in certain systems like V_2_O_5_–Zn. Further, all three cells show an initial increase followed by a decrease in capacities. This initial increase in capacity is caused by an increase in the utilization of the V_2_O_5_ electrode^35^ (activation, described in Supplementary Note 3). After complete conversion into Zn_*x*_V_2_O_5_·*n*H_2_O, the capacity of the cell stabilizes, and the cell is fully activated. Figure 2(a) shows that the cell illuminated under 1 sun and the cell placed in the incubator show a peak in capacity at approximately the same cycle number (∼30), around 30 cycles earlier than the cell at room temperature, indicating that thermal effects can accelerate the activation of V_2_O_5_.

**Figure 2:**
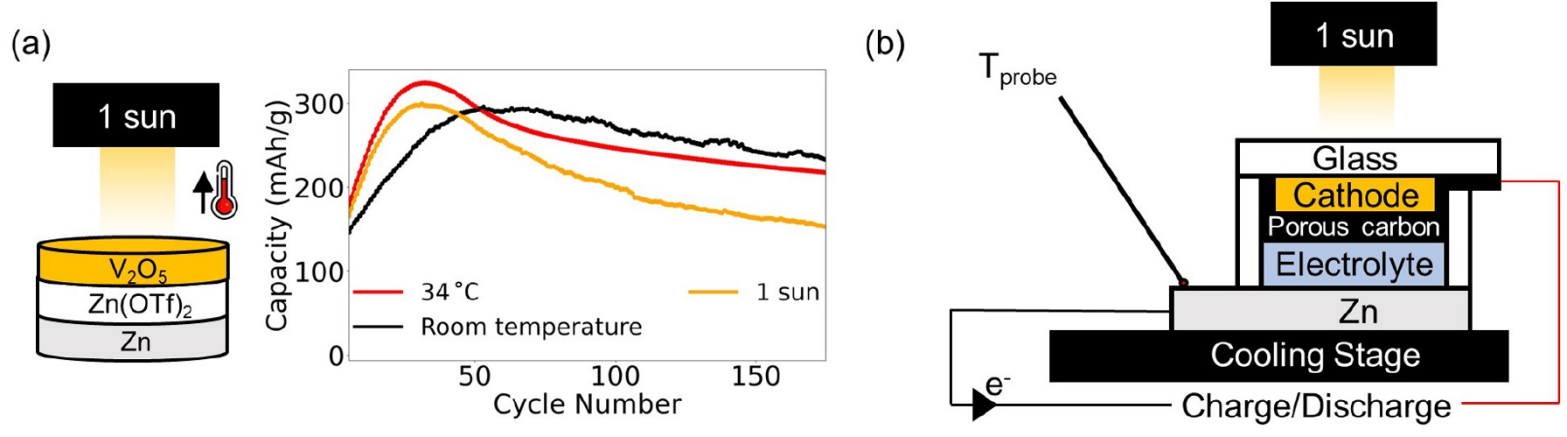
Thermal considerations while conducting experiments on light-rechargeable photobatteries. (a) Effects of heat and light on the capacity of a V_2_O_5_–Zn battery; both heat and light increase the capacity of the cell and accelerate its activation process. (b) Planar cell structure that enables simultaneous cooling of photobatteries during photoelectrochemical measurements, allowing for the thermal contributions to capacity enhancement to be subtracted.

Hence, we show that the thermal effects of solar irradiation can lead to erroneous calculations of capacity increases attributed to light interaction. This may be due to thermal reductions in the overpotential of the electrochemical processes, accelerated activation of the material, or a combination of both of these and other effects. Therefore, it is important to track the increase in the temperature of the cell during light charging experiments. However, coin cells are not suitable for this, as described in Supplementary Note 4.

To perform temperature-controlled photoelectrochemical measurements on illuminated photobatteries, a new cell design that allows for effective heat conduction is required. We propose a cell architecture (henceforth referred to as a planar cell) that is similar to those used in dye-sensitized solar cells (DSSCs) in Figure 2(b), consisting of a zinc anode and a glass window with the cathode and a carbon-based current collector tape-casted on it. We avoid using FTO due to side reactions observed when cycling between 0.2 and 1.6 V against a zinc metal anode in a 3 M Zn(OTf)_2_ electrolyte (see Figure S2 and Supplementary Note 5). The steps for fabricating a planar cell are provided in Figure S3, with the cell demonstrating a stable cycling performance over 80 cycles (Figure S4).

Next, we use these cells to focus on the optical changes in V_2_O_5_ as it is cycled. *Operando* optical microscopy is used to record changes in the optical appearance of V_2_O_5_ as a function of the state of charge vs Zn. V_2_O_5_ nanowires were prepared by using hydrothermal synthesis. A SEM image, Raman spectrum, XRD data, and UV–vis measurements of the synthesized powder are shown in Figure S5. The color of a material includes contributions from both its band structure and defects. Therefore, tracking the appearance of a photocathode can reveal valuable information about where changes in band structure may occur as a function of the state of charge. The results are shown in Figure 3 and Supplementary Video 1. The color of the photocathode starts off as yellow and turns gray as the cell is discharged. During the charging process, the electrode starts to turn yellow at a potential of 1.2 V before regaining its color when fully charged (1.6 V). This change is further represented in Figure S6, where we plot the hue^36^ of a subsection of the image as a function of the state of charge, with a clear reversible trend seen. This indicates that the intercalation of zinc ions can change the optical properties and the electronic structure of V_2_O_5_ (as seen in Li–V_2_O_5_ as well^37^), with most changes occurring between 0.8 and 1.2 V. The discrepancy in the color of the cell before and after cycling is due to the slight self-discharge of the cell during overnight resting after electrolyte injection.

**Figure 3:**
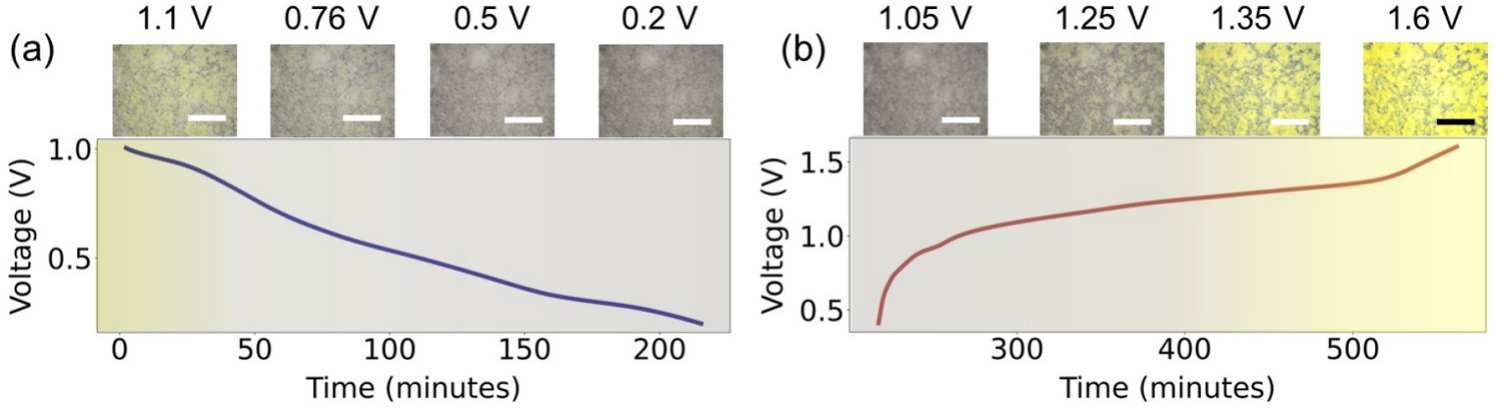
*Operando* optical microscopy images of a cell (a) discharged to 0.2 V and (b) charged to 1.6 V. A change in color from yellowish to gray is seen at a discharge voltage of 0.8 V; this color is retained until the cell is charged to 1.25 V, at which point the yellowish color returns. This indicates that the optical and electronic properties of the system are changed as the zinc ions are intercalated. However, this change is at least partly reversible, as indicated by the return of color in the photocathode as ions are de-intercalated. All scale bars represent 400 μm.

To quantify changes in the optical properties of V_2_O_5_ upon intercalation, we use *operando* reflection spectroscopy, as shown in Figure 4. An integrating sphere is coupled with a UV–vis spectrometer to enable accurate measurements of diffuse reflectance spectra from the cathode during electrochemical cycling. Our planar cell design allows for easy mounting on the integrating sphere (as shown in Figure S7). We describe the utility of using in situ reflection spectroscopy to track optical changes in a photocathode in Supplementary Note 6.

**Figure 4:**
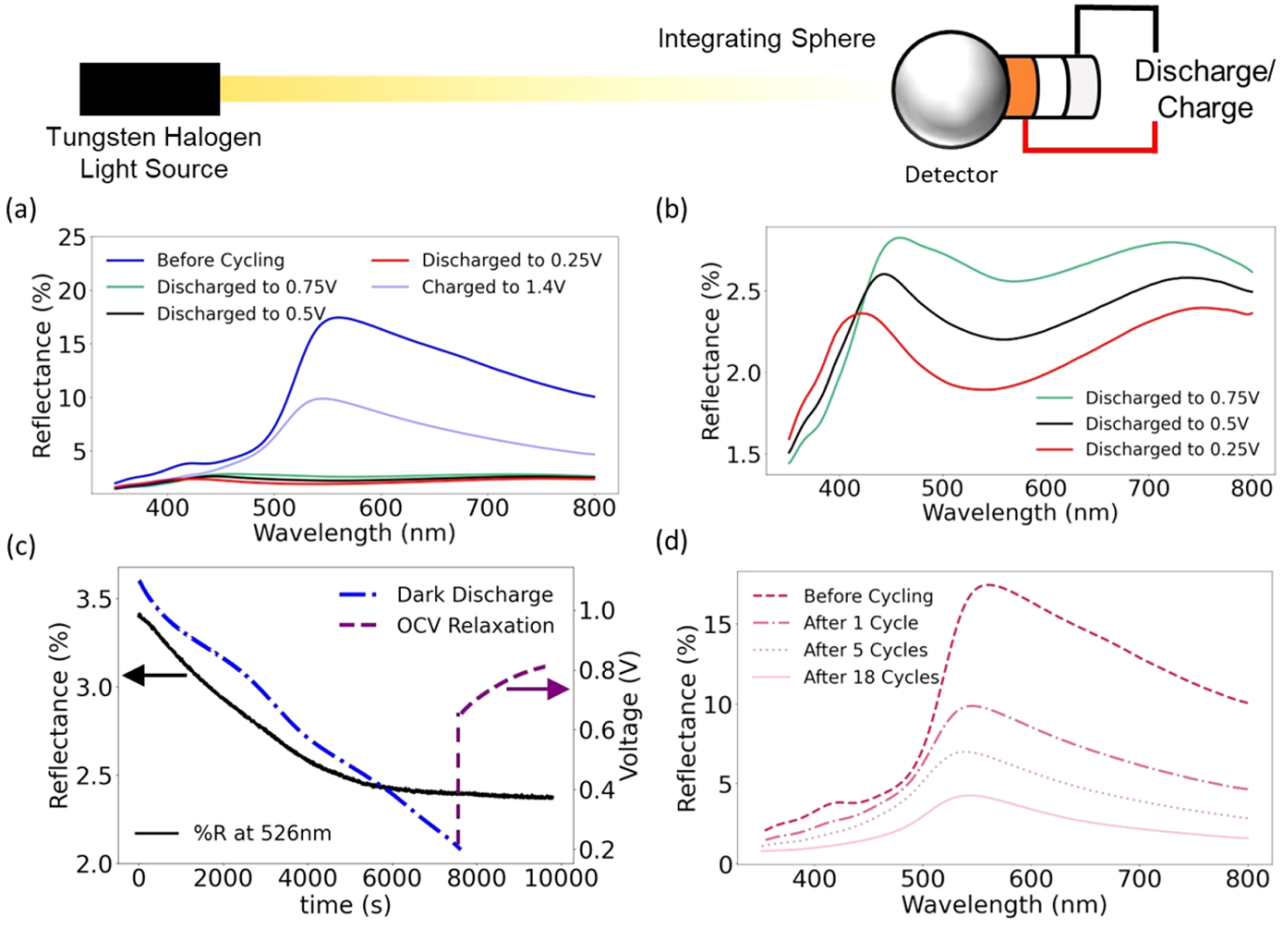
Changes in the reflectance of V_2_O_5_ when cycled against a zinc anode. (a) *Operando* reflection spectra of V_2_O_5_ photocathodes cycled against a Zn metal anode for different discharge voltages. The discharge protocol is provided in Figure S8. A clear reflection edge at about 520 nm (indicating the onset of the band gap) is seen before the cell is cycled and after it is charged to 1.4 V. (b) Enlarged reflection spectra for the discharged photocathode seen in (a) at 0.75, 0.5, and 0.25 V. The magnitude of reflection is significantly diminished and the reflection edge has shifted to about 400 nm, indicating a significant change in optical properties due to intercalation. (c) Reflectance at 526 nm measured while the cell is galvanostatically discharged. The reflectance decreases continuously as ions are intercalated, before flatlining at 0.38 V. The purple line tracks the reflectance of the cell while it is resting under OCV conditions. (d) Reflectance spectra of cells after 1, 5, and 18 cycles, showcasing a continued decrease in the magnitude of reflectance when cycled between 0.2 and 1.6 V.

We discharge the planar cell mounted on the integrating sphere to 0.75, 0.5, and 0.25 V, charge the cell to 1.4 V, and record the reflectance spectra at each point as shown in Figure 4(a). The discharging/charging protocol is shown in Figure S8. Due to the low amount of conductive additive present in the cathode (1% to prevent excessive light absorption) and the thick drop-cast photocathode, the overpotentials associated with the cell are quite high. Hence, we apply 2 h voltage holds at each charge/discharge potential to ensure that the entire cathode equilibrates to the same state of charge. Before cycling, the spectrum shows a sharp increase in reflectance, culminating in a peak at 526 nm. This closely matches the band gap of V_2_O_5_, as determined from absorbance measurements in solution (Figure S5). After the cell is discharged to 0.75, 0.5, and 0.25 V, the magnitude of the reflectance is significantly reduced by a factor of ∼8 (an enlarged image of this is shown in Figure 4(b)). The reflectance edge also shifts to ∼420–450 nm, indicating a change in the optical properties of the material. When the material is charged back up to 1.4 V (de-intercalation), the reflectance spectra increase in magnitude, and the reflectance edge reappears with a peak at 545 nm. The decrease in the magnitude of reflectance is consistent with the aforementioned activation effect in V_2_O_5_ due to the complete conversion of the cathode into Zn_*x*_V_2_O_5_·*n*H_2_O, which results in an irreversible shift in optical properties. However, other effects such as salt deposition or the formation of electrochemically inactive zinc pyrovanadate^38^ may also contribute to this decrease in reflectance.

Figure 4(c) tracks the reflectance of the cathode at 526 nm (the initial reflectance peak) during a discharge cycle (to 0.2 V). The reflectance continuously decreases as the cell is discharged, flatlining at 0.38 V. This suggests that continued zinc ion intercalation results in a decrease in the reflectance of V_2_O_5_ until a certain point, after which the optical properties do not change. Discharging the cell may lead to the degenerate doping limit of the semiconductor being reached, as described in Supplementary Note 7, after which the material will not have a band gap.

When zinc-ion batteries based on vanadium cathodes are fully discharged and allowed to rest, their OCV has been reported to increase during the resting phase, even though no external potential is applied.^39^ When the cell is allowed to relax for 40 min, the OCV of the cell rises; however, no change in reflection is seen, suggesting that the state of charge of the cell does not change over the time scale studied. Finally, Figure 4(d) shows reflection spectra of the cell at its equilibrium (open-circuit) voltage before cycling and after 1, 5, and 18 cycles. A continued decrease in the magnitude of the reflectance is consistent with the fact that the V_2_O_5_ cathode is increasingly converted to Zn_*x*_V_2_O_5_·*n*H_2_O over the first few cycles. Therefore, it appears that continued intercalation is detrimental to the optical response of a photocathode, which is a key consideration while identifying future candidates for photocathodes.

Next, we designed a control experiment to study thermal effects in photobattery cells using cyclic voltammetry. We constructed two kinds of planar cells—one with the active material facing the solar simulator and one where the carbon film blocks light from interacting with the active material (Figure S10). The transmittance spectrum of the carbon film is shown in Figure S9, indicating that no light–cathode interactions occur in the latter. Any enhancements in capacity or reduction in overpotentials in the second case should therefore solely be due to thermal effects. We test this control experiment on two photocathodes, V_2_O_5_ and LiMn_2_O_4_. Our V_2_O_5_ photocathode consists of a mixture of V_2_O_5_, P3HT, and rGO, as these additives have been reported to act as efficient electron-transport layers.^7^ Although LiMn_2_O_4_ is typically cycled against a lithium counter electrode, recent advances in dual-ion water-in-salt electrolytes (WiSEs)^40^ have enabled cycling against a zinc counter electrode, allowing for easier fabrication of planar cells simultaneous with cell cooling, as shown later. Information about the cell construction and electrochemistry can be found in Supplementary Note 8 and Figure S12.

The results are shown in Figure 5. To establish a baseline capacity, we initially cycled the cells in the dark (20 cycles for V_2_O_5_–Zn and 15 cycles for LiMn_2_O_4_–Zn) prior to illumination. When illuminated, both cell types (facing and not facing light) and both active materials show an increase in the area under their CV curves. Comparing cells where the active material faces or is blocked from illumination, the respective increases are 22% and 26% for V_2_O_5_ (Figure 5(a) and (b)) and 9% and 7% for LiMn_2_O_4_ (Figure 5(c) and (d)). This indicates, for both cells, that thermal effects can contribute almost entirely to increases in capacity. This could be due to reductions in impedance at higher temperatures, as demonstrated earlier. Figure 5(e)–(h) demonstrates that the increase in capacity can be sustained over several cycles for both materials in both cell configurations, indicating that thermal effects can affect long-term cycling performances.

**Figure 5:**
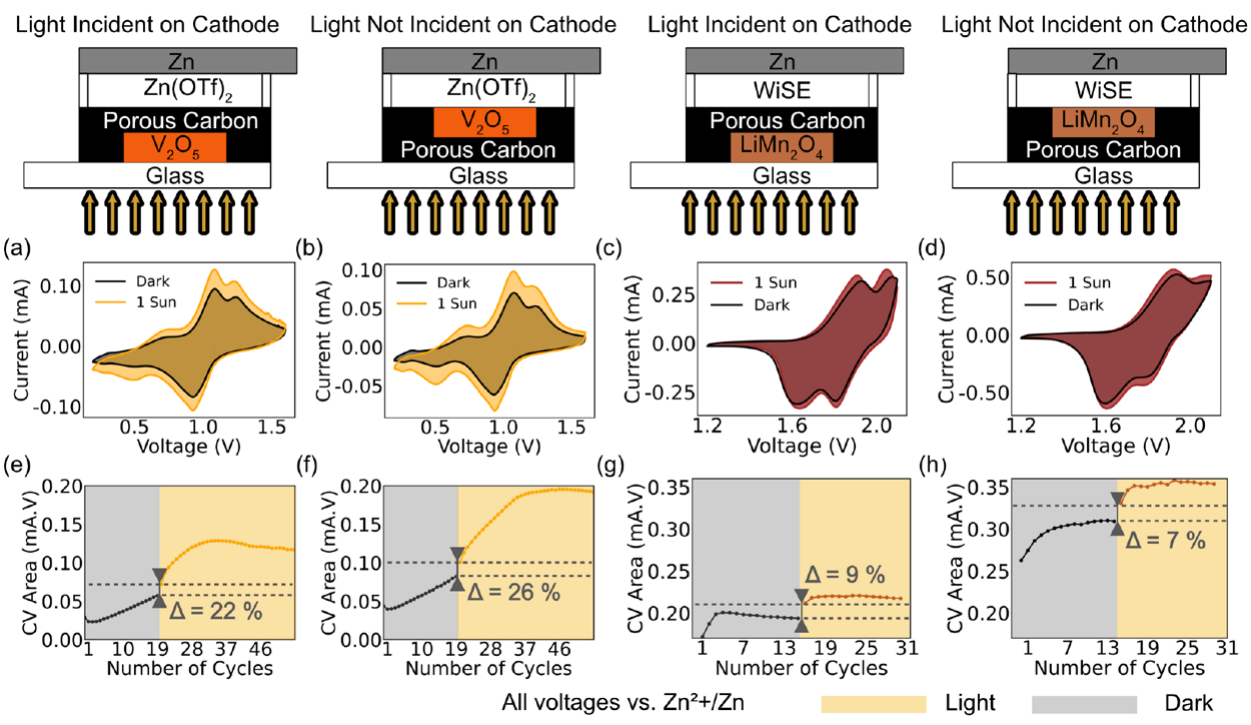
CV curves under dark and illuminated conditions for V_2_O_5_–Zn and LiMn_2_O_4_–Zn cells. (a) The active material in the cell is facing the illumination source, and (b) the active material is shielded from the light source by a layer of carbon. A similar experiment is performed for LiMn_2_O_4_–Zn cells, where (c) the active material is illuminated and (d) the active material is obscured by a layer of carbon. For both V_2_O_5_ (dark cycle 20 and light cycle 21 in (e) and (f)) and LiMn_2_O_4_ (dark cycle 15 and light cycle 16 in (g) and (h)), the area under the CV curves shows a very similar increase, regardless of whether the active material is facing or shielded from the light (22% vs 26% for V_2_O_5_ and 9% vs 7% for LiMn_2_O_4_).

To isolate genuine optical effects, a method for subtracting thermal contributions to capacity enhancement is needed. By placing the anode of a planar cell directly on a cooling stage (as shown in Figure S10), we allow for effective thermal dissipation of heat generated from solar irradiation, as the electrolyte is in direct contact with the anode. Using an IR temperature gun [KKMoon G300], we measure the temperature difference between the anode and cathode to be less than 1 °C, which indicates a uniform temperature difference across the cell; however, it must be cautioned here that IR guns are rarely used for precise measurement of temperature (to within a few degrees). We demonstrate the utility of thermal controls on LiMn_2_O_4_–Zn cells in Figure 6, where the steps represented were carried out sequentially. Initially, we charge and discharge the cell under dark conditions, as shown in Figure 6(a) and (c) (Steps 1 and 2), to establish a baseline for the cell. Next, we charge the cell under a 1 sun condition (Step 3). A decrease in charging overpotentials is seen, and the discharge capacity rises from 112.6 to 114.5 mAh g^–1^ (Step 4). However, when we charge the cell by placing the zinc anode on a stage heated to 32 °C, the temperature of the cell recorded under 1 sun illumination in the previous charge (Step 5), we see a similar rise in discharge capacity to 114.8 mAh g^–1^ (Step 6). This suggests that a large proportion of the capacity enhancements seen could be due to thermal effects.

**Figure 6:**
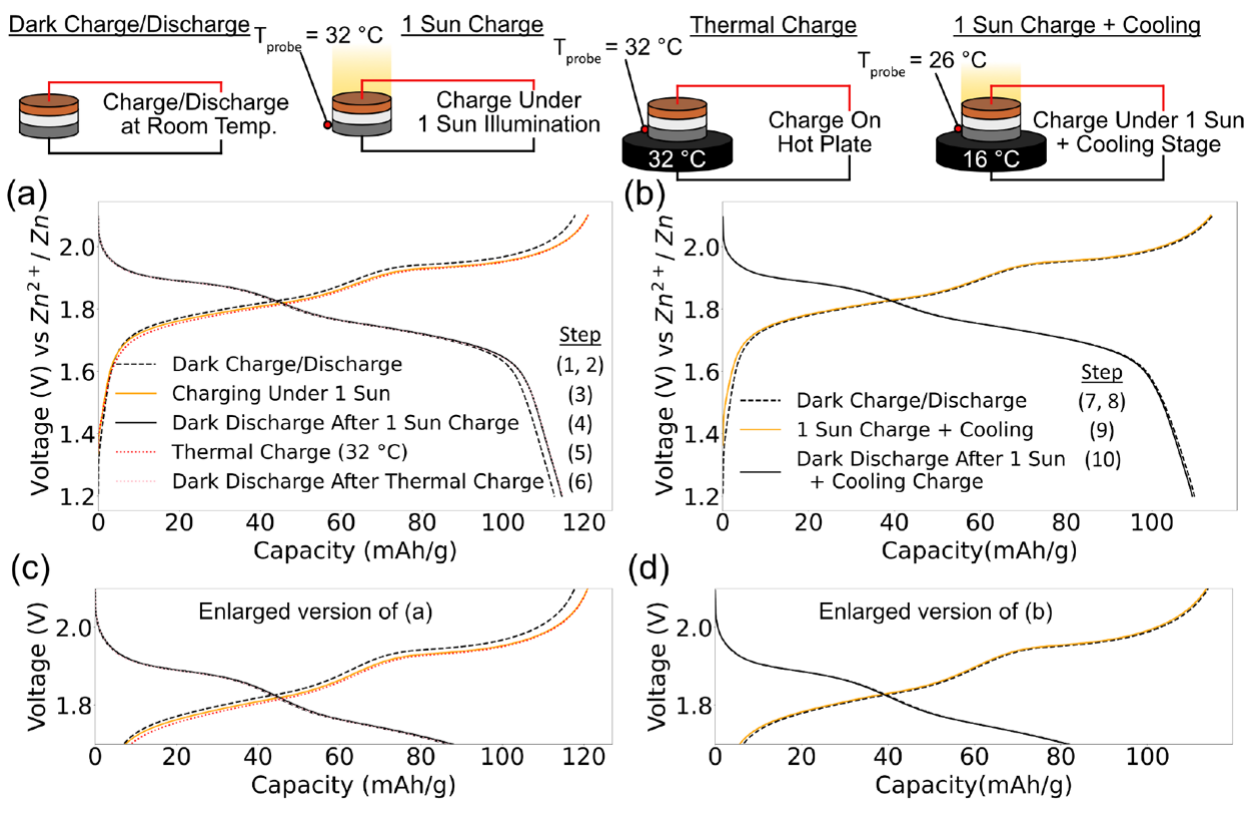
Thermal controls on LiMn_2_O_4_–Zn cells. (a) A planar LiMn_2_O_4_–Zn cell was initially charged (Step 1) and discharged (Step 2) in the dark (no heating or illumination) to establish a baseline capacity. Next, the cell was charged under 1 sun illumination (Step 3) and discharged in the dark (Step 4). The temperature probe records a temperature of 32 °C. Then, the cell was charged while maintaining a probe temperature of 32 °C (Step 5) and discharged (Step 6). (b) The cell was charged and discharged in the dark (Steps 7 and 8) and then charged under 1 sun illumination while cooling to maintain the initial temperature of the cell (Step 9), followed by discharge under ambient conditions (Step 10). After subtraction of thermal effects, the discharge capacity of the cell is very similar to its original baseline. Panels (c) and (d) are enlarged versions of (a) and (b), respectively, in the voltage range of 1.7–2.1 V.

Next, we investigated whether simultaneously cooling the cell can subtract these thermal contributions. We placed the zinc anode on a cooling plate under 1 sun irradiation, as shown in Figure 6(b) and (d). The temperature of the cooling stage was adjusted (to 16 °C) until the temperature of the probe was equal to the temperature recorded under ambient conditions before the experiment was started (26 °C). The cell was then charged when equilibrium between the incident radiative heat from the solar simulator and thermal conduction from the stage was reached (Step 9). The cell shows a baseline discharge capacity of 109.9 mAh g^–1^ in the dark (Step 8) and 109.4 mAh g^–1^ when cooled under 1 sun irradiation (Step 10), which shows that the capacity enhancements seen (Figure 5(a)) were primarily due to thermal effects. A demonstration of how the experiments were carried out is provided in the temperature profile in Figure S13, along with a summary of recorded capacities in Table S1 and Table S2. Figure 6(c) and (d) are enlarged versions of (a) and (b). We also performed chronoamperometry (CA) measurements, as shown in Figure S14 and Supplementary Note 9, along with experiments on the contributions of infrared heating described in Table S3, Figure S15, and Supplementary Note 10.

We next focus on whether the V_2_O_5_–Zn with a P3HT-PCBM electron transport layer (see Figure S11 for the energy diagram) can be photocharged. Our results are described in Figure S16 and Supplementary Note 11, indicating that the system cannot be directly photocharged and can only be operated in photoassisted modes. Instead, several publications^5,7,10,24,33^ report carrying out photocharging in open circuit or under a high resistive load,^6^ leading to an increase in voltage under illumination (henceforth referred to as OCV charging). As no electrons can flow under open-circuit conditions, the increase in voltage must occur due to a single-electrode process similar to those described previously.^39^ Additionally, this is not demonstrated for all electrochemical systems: when the LiMn_2_O_4_–Zn is illuminated under OCV conditions, a decrease in OCV is seen rather than an increase, as shown in Figure S17. Some publications have attributed this stored energy to the formation of a double layer on the surface of photocathode particles.^41^ Nevertheless, OCV charging represents an interesting way to acquire energy from an electrochemical system. However, here we show that thermal effects can also influence the discharge capacity, which can be attained from the cell. The thermal charging of electrical double-layer capacitors has been reported earlier^42^ and was attributed to enhanced kinetics for sluggish Faradaic reactions at higher temperatures.

We performed the OCV charging experiments on V_2_O_5_–Zn cells as shown in Figure 7. In Figure 7(a) the cell is discharged to 0.2 V, followed by a voltage hold for 2 h at 0.2 V to discharge the cell fully. A discharge capacity of 0.29 mAh is recorded. However, as soon as the voltage hold is completed, a rise in the OCV of the cell is seen. After 30 h, the OCV reaches a plateau at 0.67 V. When the cell is discharged from this point to 0.2 V, a capacity of 0.06 mAh is obtained. Next, the cell is allowed to relax under 1 sun illumination for 26 h. The OCV of the cell rises again to a value of 1.056 V. When the cell is discharged to 0.2 V, a discharge capacity of 0.1 mAh is recorded. After this, the cell is allowed to relax while being heated at 34 °C in an incubator, which was the highest temperature recorded under 1 sun illumination.

**Figure 7:**
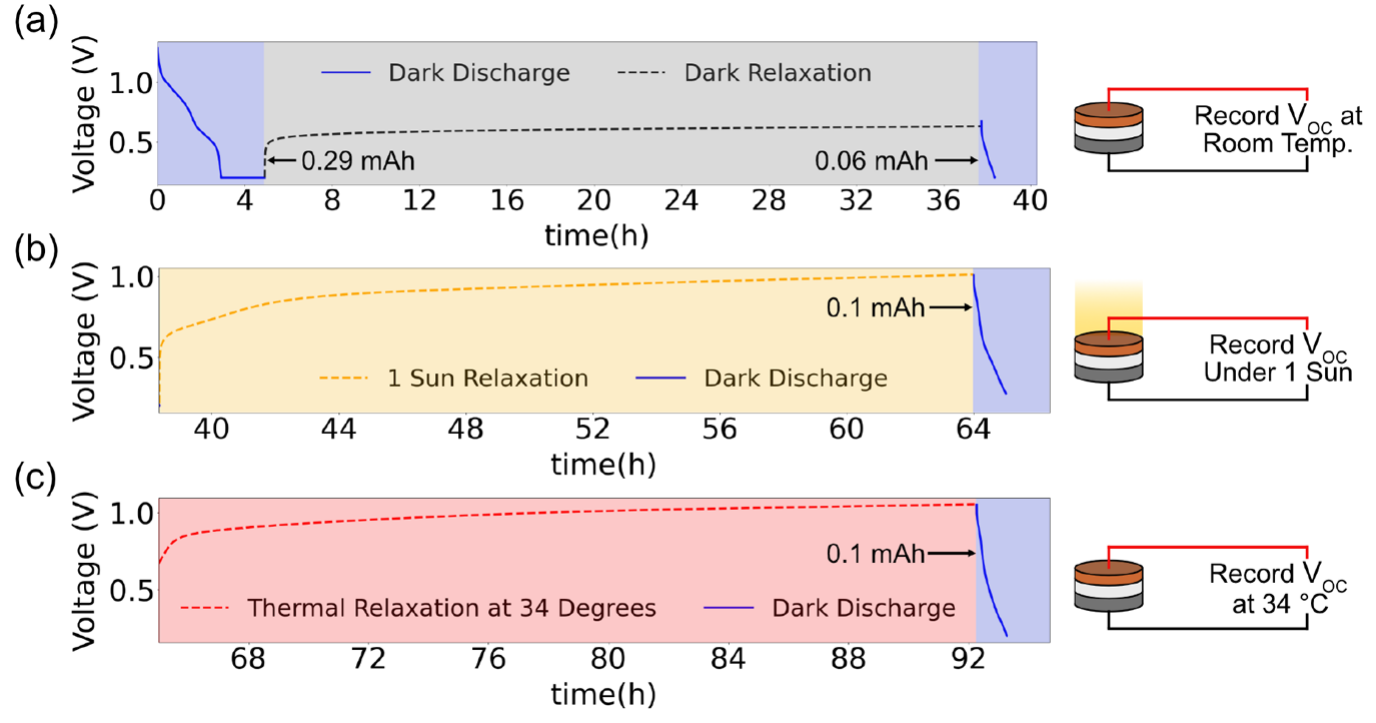
Decoupling thermal and light effects under OCV charging for a V_2_O_5_–Zn cell. (a) Voltage vs time plots are shown for a cell that was initially discharged to a voltage of 0.2 V and displayed a discharge capacity of 0.29 mAh. The cell was allowed to relax in the dark for 32 h and then discharged again. A discharge capacity of 0.06 mAh was recorded. (b) The cell was subsequently allowed to relax under 1 sun illumination for 26 h. A rise in OCV to 1.056 V is measured. The cell is then discharged to 0.2 V, and a discharge capacity of 0.1 mAh is observed. (c) The cell is then allowed to relax in an incubator for 27 h. The OCV of the cell rises to 1.045 V. The cell is then discharged to obtain a discharge capacity of 0.1 mAh.

A similar rise in the OCV of the cell is seen (to 1.045 V) after 27 h of relaxation. When the cell is discharged after the thermal relaxation, a discharge capacity of 0.1 mAh is seen, which is close to the value recorded under 1 sun illumination. This suggests that thermal effects also play a large role in the discharge capacities of the OCV-charged cells. Therefore, a cell design that allows for simultaneous cooling of generated heat is crucial for the reliable measurement of photoenhanced capacities during both illuminated galvanostatic measurements and OCV charging.

In conclusion, we highlight that when a cell is illuminated, the resultant capacity enhancements can be due to both thermal and optical effects. To report such enhancements accurately, an effective way to decouple these phenomena is needed. We demonstrate the importance of the positions of the electron and hole quasi-Fermi levels of the photocathode relative to the cathode and anode de-intercalation and plating potentials. Using *operando* reflection spectroscopy and thermal controls on two promising photocathodes, V_2_O_5_ and LiMn_2_O_4_, in aqueous electrolytes, we track changes in the optical properties of photocathodes upon intercalation. It is shown that continued intercalation of zinc ions into V_2_O_5_ results in a loss of band gap for the material, making it unsuitable for use as a photocathode at a voltage below 0.75 V. We also show that the thermal effects of 1 sun irradiation can result in enhanced capacities either due to accelerated activation of the material or due to a rise in the temperature of the battery. We demonstrate a new planar cell architecture that is compatible with setups allowing for the simultaneous cooling of the cell, allowing thermal effects to be subtracted. Through this, we show that the resultant increases in capacity upon illumination in both of the photocathodes studied are almost entirely due to thermal effects. We believe that these results will establish guidelines for material selection for next-generation photobatteries while allowing for the reliable reporting of their photoenhanced behavior.

## Supporting Information Available

Materials and methods, additional photoelectrochemical measurements, pictures of setup, material characterization, optical image analysis, EIS data, tables with photoelectrochemical data, and notes with additional experimental and theoretical details (PDF)
Supplementary Video 1, showing changes in the optical appearance of V_2_O_5_ as a function of the state of charge vs Zn (AVI)

## Supplementary Material

nz3c01627_si_001.pdf

nz3c01627_si_002.avi
