# Supplementary material for: Does Heat Play a Role in the Observed Behavior of Aqueous Photobatteries?
Source: ACS Energy Lett. 2023 Oct 12;8(11):4625–33. doi: 10.1021/acsenergylett.3c01627 (PMC10644369; doi:10.1021/acsenergylett.3c01627)
Supplement: Supplementary file 1 — nz3c01627_si_001.pdf [file nz3c01627_si_001.pdf]

Supplementary Material for 'Does Heat Play a Role in the Observed Behavior of Aqueous Photobatteries?'

Arvind Pujari[1, 2], Byung-Man Kim[2], Farheen N. Sayed[3], Kate Sanders[2], Wesley M. Dose[2, 3, 4], Angus Mathieson[2], Clare P. Grey[3], Neil C. Greenham[1], and Michael De Volder[2,*]

[1]Cavendish Laboratory, Department of Physics, University of Cambridge, Cambridge, CB3 0HE, UK

[2]Institute for Manufacturing, Department of Engineering, University of Cambridge, Cambridge, CB3 0FE, UK

[3]Department of Chemistry, University of Cambridge, Cambridge, CB2 1EW, UK

[4]School of Chemistry, University of New South Wales, Sydney, NSW, AU 2052

[*]Address correspondence to mfld2@cam.ac.uk

1 Experimental

1.1 Material Synthesis and Characterization

V_2O_5 nanofibres were synthesized through a hydrothermal process. 1.46 g of V_2O_5 powder (Sigma Aldrich, 98%) was mixed in 120 ml water under vigorous stirring. Then, 20 ml of H_2O_2 (30%, Sigma Aldrich) was added drop by drop under stirring until a transparent orange solution was obtained. The solution was then transferred into a 120ml teflon autoclave and heated at 200 °C for two days. The product was washed several times with water and ethanol and then annealed at 400 °C in air.

X-ray powder diffraction (XRD) data of the sample was collected using a Bruker D8 Advance X-ray diffractometer (Cu Kα radiation). Raman spectra were obtained using a Reinshaw InVia Raman Microscope (532 nm laser) while scanning electron microscope (SEM) images were collected using a FEI Dualbeam Quanta 3D. UV-vis absorption and reflection measurements were carried out using a UV/VIS/NIR Spectrometer (Perkin Elmer Lambda 750) coupled with a 100 mm integrating sphere. A Spectralon diffuse reflectance standard was used as a reference for all reflectance measurements. Spectra were passed through a Savitzky-Golay filter prior to plotting to reduce noise. Optical microscopy images were taken using a BX53M (Olympus) combined reflected/transmitted light microscope with an LC30 camera coupled with an autofocus setup. Both *operando* reflection spectroscopy and optical microscopy measurements were carried out during galvanostatic cycling of the cell using an Autolab PGSTAT204.

V_2O_5 electrode solutions were prepared by dispersing 93 mg of the as obtained V_2O_5 nanofibres, 1 mg of poly(3-hexylthiophene) (P3HT, Osilla) and 1 mg reduced graphene oxide (rGO, Sigma Aldrich) in 4 ml of N-methyl-2-pyrrolidone (NMP, Sigma Aldrich) followed by mixing with 5 mg of polyvinylidene fluoride (PVDF, Solef 6020)

binder. For photocharging experiments on Zn-V_2O_5 the same electrode solution was used but with 0.5 mg of P3HT and 0.5 mg of PCBM ([6,6] phenyl-C61-butyric acid methyl ester, Osilla) as the electron transport layer instead.

Lithium manganese oxide ($LiMn_2O_4$) nanopowders were obtained from Sigma Aldrich. Electrode solutions were obtained by grinding 80 mg $LiMn_2O_4$ and 10mg Super P (Tincal, MTI) in a mortar and pestle before mixing with 10 mg PVDF (Solef 6020) and 400 μL of NMP in a planetary mixer for 20 minutes.

1.2 Deposition of LMO Thin Films

For thin film deposition, an LMO target was prepared by ball milling LMO with 10 wt% excess Li_2O in zirconia ball mill jar for 1 hour. The obtained powder was pelletised and sintered at 900 ^{o}C in air for 10 hours. The sintered pellet was then used for deposition of LMO thin films in a pulsed laser deposition (PLD) system equipped with a KrF excimer laser (wavelength = 248 nm). After placing the target and pre-cleaned substrate inside, the deposition chamber was brought to a base pressure of 10^{-5} Pa. The optimised conditions used for the deposition were: T = 500 °C, F (Fluence) = 2.0 J cm^2 , p_{O_2} = 0.3 hPa, ν (Pulse Frequency) = 10 Hz, Z_{height} = 45 mm and deposition shots = 36000. The deposition was followed by the annealing of film inside the same chamber at 550 ^{o}C with 10 hPa of oxygen for 30 minutes.

1.3 Fabrication of Photobatteries

To make coin cells, 20 μL of the as-prepared electrode solution was dropcast on 10 mm disks of carbon paper (SGL Carbon) which had been subjected to 1 hour of ultraviolet (UV) - ozone treatment to improve its hydrophilicity. The substrates were then dried for 1 hour on a 120 °C hotplate. Coin cells (CR2040) with 8 mm drilled holes were used for photoelectrochemical measurements. A glass coverslip (1.1 mm) cut in the shape of a circle and stuck using epoxy (EVO-STIK) was used as an optical window.

The carbon paper was placed on the glass and electrical connections to the can of the coin cell were made using carbon nanotubes (CNT) paper (Tortech, 50 µm). A few drops of electrolyte (3M $Zn(OTf)_2$, Alfa Aesar in deionized (DI) water, where OTf = triflate) were dropped onto the photocathode and then a Whatmann glass fibre separator (GF/B, 19mm) was placed on it along with 200 µl of electrolyte. A metallic zinc foil (Alfa Aesar, 0.25 mm) was used as the counter electrode. The coin cells were then sealed by crimping at a pressure of 1000 psi.

To make planar cells where the cathode material can be optically illuminated, two 1 mm holes were drilled on a pre-cut glass coverslip (1.1 mm) using a Dremmel workstation. The glass was then cleaned by sonication in acetone (40 minutes), isopropyl alcohol (IPA, 10 minutes), and water (10 minutes). 5 µL of the as-prepared electrode solution was dropped on the glass which was then dried for 1 hour on a 120 °C hotplate. Carbon paste (Dycotec, DM-CAP-4701S) was deposited on top of the cathode by tape casting (tape thickness of 60 µm) followed by 30 minutes of drying at 75 °C. To assemble the cell, 4 rings of thermoplastic sealant (Surlyn, Greatcell SKU MS004610-0) were placed around the cathode material. A hydrophilic polyvinylidene fluoride (PVDF, Thermofisher) membrane was placed on top of the cathode as a separator. A 0.25 mm zinc foil (Alfa Aesar) was placed on top of the thermoplastic and the entire device was sealed on a 120 °C hotplate by applying pressure until the thermoplastic was melted. The electrolyte used was 3M $Zn(OTf)_2$ in DI water for the V_2O_5-Zn system and 20 mol g^{-1} LiTFSI (Sigma Aldrich) + 1 mol g^{-1} $Zn(TFSI)_2$ (Alfa Aesar) in DI water for the $LiMn_2O_4$-Zn system. The electrolyte was injected into the cell using the pre-drilled holes which were then sealed using UV-curable epoxy (Norland Optical Adhesive NOA 61). Finally, electric contacts were soldered to the carbon paste using an ultrasonic soldering machine (Sunbonder USM-560).

To make a planar cell in which the cathode material was not facing the light, the

same steps as above were followed except that first a carbon layer was deposited on glass using tape casting followed by the dropcasting of 5 μL of the as-prepared electrode solution and drying, effectively inverting the optically opaque carbon layer and photoactive layer.

1.4 Electrochemical Characterization

A Biologic VMP-3 galvanostat was used for all electrochemical characterization except *operando* experiments. A solar simulator (Newport Scientific LSH-7320) with an intensity of 1 sun (100 mW cm^{-2}) was used to illuminate the photobatteries. Cyclic Voltammetry (CV) scans were carried out at 1 mV s^{-1}. Galvanostatic charge and discharge measurements were carried out at 1C under dark and illuminated conditions (where 1C = discharge capacity obtained at a current density of 14.8 $\mathrm{m\,A\,g^{-1}}$ which is 120 $\mathrm{m\,A\,h\,g^{-1}}$. The solar simulator was switched on during the charging process and switched off while discharging. The voltage range used was 0.2-1.6 V for V_2O_5-Zn batteries and 1.2 - 2.1 V for $LiMn_2O_4$-Zn batteries. Thermal controls of coin cells were carried out in an incubator at 34 °C (Sciquip). OCV charging measurements were performed by recording the change in the OCV of the cell under dark, 1 sun, and heated conditions. Electrochemical impedance spectroscopy (EIS) spectra were recorded from 10 kHz to 10 mHz with an amplitude of 10 mV. For fitting EIS specta, a general resistance/CPE pair (R_w/C_w) was used instead of Warburg element because the spectra has mid shape between general capacitance and Warburg diffusion line.

1.5 Temperature Controls

All cells were mounted with the anode (zinc foil) on a cooling stage (Thorlabs, PTC1/M) and pressed down using adhesive tape (3M) to establish good thermal contact. The same stage could also be used to heat the cell to the desired temperature. A separate thermocouple (Thorlabs TSP01) was placed on the zinc anode to monitor its temperature directly. To cool the cell during photoelectrochemical measurements, firstly, the

initial temperature was recorded. After the solar simulator was switched on a rise in temperature was recorded which was countered by setting the temperature of the cooling stage to 15-17 °C until the temperature stabilized to a value close to that recorded initially.

2 Supplementary Notes, Tables, and Figures

Condition	$C_{charged}$ (mAhg^{-1})	$C_{discharged}$ (mAhg^{-1})	CE (%)
Dark	117.7	112.6	95.7
1 sun	120.9	114.5	94.7
Heated (32 °C)	121.0	114.8	94.9

Table S1: Charge and discharge capacities and coloumbic efficiencies for $LiMn_2O_4$-Zn cells charged and discharged in the dark, charged under 1 sun illumination and discharged in the dark, and, charged under heated conditions and discharged under ambient conditions.

Condition	$C_{charged}$ (mAhg^{-1})	$C_{discharged}$ (mAhg^{-1})	CE (%)
Dark	114.1	109.9	96.3
1 sun + Cooling	113.7	109.4	96.2

Table S2: Charge and discharge capacities and coloumbic efficiencies for $LiMn_2O_4$-Zn cells charged and discharged in the dark and charged under 1 sun illumination with simultaneous cooling and discharged in the dark. The difference in the dark discharge baseline capacities between Table S1 and Table S2 is due to differences in temperatures of the room on the days where the experiments were conducted.

Condition	$C_{charged}$ (mAhg^{-1})	$C_{discharged}$ (mAhg^{-1})	CE (%)
Dark	109.3	106.0	97.0
1 sun + IR filter	113.1	106.9	94.5
1 sun and no IR filter	114.1	107.4	94.1

Table S3: Charge and discharge capacities and coloumbic efficiencies for $LiMn_2O_4$-Zn cells charged and discharged in the dark and charged under 1 sun illumination with and without an IR filter. The differences in dark capacity between Tables S3 and Tables S1/S2 are due to a different cell with slightly different mass loading being used.

Supplementary Note 1 - Band alignment considerations in photobatteries: In photobatteries with a metallic anode, electrons must be the majority carrier to ensure zinc reduction at the anode. Therefore, the semiconductor used should also be n-type for photocharging to proceed effectively. In practice, E_{Fv} and E_{Fc} are close to the valence band positions and conduction band positions and can be measured by ultraviolet photoelectron spectroscopy (UPS). However, depending on the synthesis method and treatment conditions the position of the conduction band can be very different. For example, Zhang et al. [1] reported that the conduction band position of V_2O_5 is -3.19 eV vs vacuum when the film is annealed at 100 °C, -3.07 eV vs vacuum when the film is annealed at 150 °C and -3.7 eV vs vacuum when the film is annealed at 180 °C. These values are measured under ultra-high vacuum conditions and are likely to change further when in contact with an acidic electrolyte.

Supplementary Note 2 - Elucidating the thermal contributions of the illumination of photocathodes: Most reported photocathodes have a band gap between 2-2.5 eV (corresponding to absorption between 500 and 650 nm), however, the solar spectrum is spread between 300-2500 nm. Thus, the excess energy of any photons which possess energy greater than the band gap is converted into heat in the form of phonons which can further contribute to the heating of the photocathode through the photothermal effect [2, 3]. 49% of sunlight is in the infrared regime (700 nm - 1 mm) which is not energetic enough to excite a semiconductor across its band gap but can contribute to the heating of the material. The equilibrium temperature a cell will achieve under illumination is dictated by balance between heating by radiation and cooling by conduction (mainly through the cell contact tabs) and convection, which is defined by the experimental setup and further complicates comparison between different publications. Overall, this indicates that a careful decoupling of thermal and photo-induced effects is required while reporting capacity enhancements during illumination.

Supplementary Note 3 - The activation process in Zn-V_2O_5 batteries: The activation process of V_2O_5 has been studied previously in literature. Zhang et. al [4] attributed this to a gradual phase change of the V_2O_5 cathode into $Zn_xV_2O_5.nH_2O$ due to irreversible zinc ion and water intercalation. This stabilizes the lattice by enlarging the interlayer spacing, allowing for higher capacities. In the same paper (Supplementary Figure 11 of that paper) it is reported that the activation time frame is shortened from 20 cycles at room temperature to 7 cycles at 50 ^{o}C which is in agreement with our experiments.

Supplementary Note 4 - Issues with temperature controls in coin cells: The presence of a window means that a small volume of electrolyte (<200 μL for coin cells), as well as the cathode, can be heated up rapidly. A coin cell has several other components like a spacer, spring, and separator which impede effective thermal dissipation by conduction from the electrolyte and the cathode. The casing of coin cells is made out of stainless steel which is one of the poorest metallic thermal conductors (thermal conductivity around 15 $WK^{-1}m^{-1}$). Therefore, merely controlling the temperature of the exterior of the cell may not result in effective management of the cell temperature.

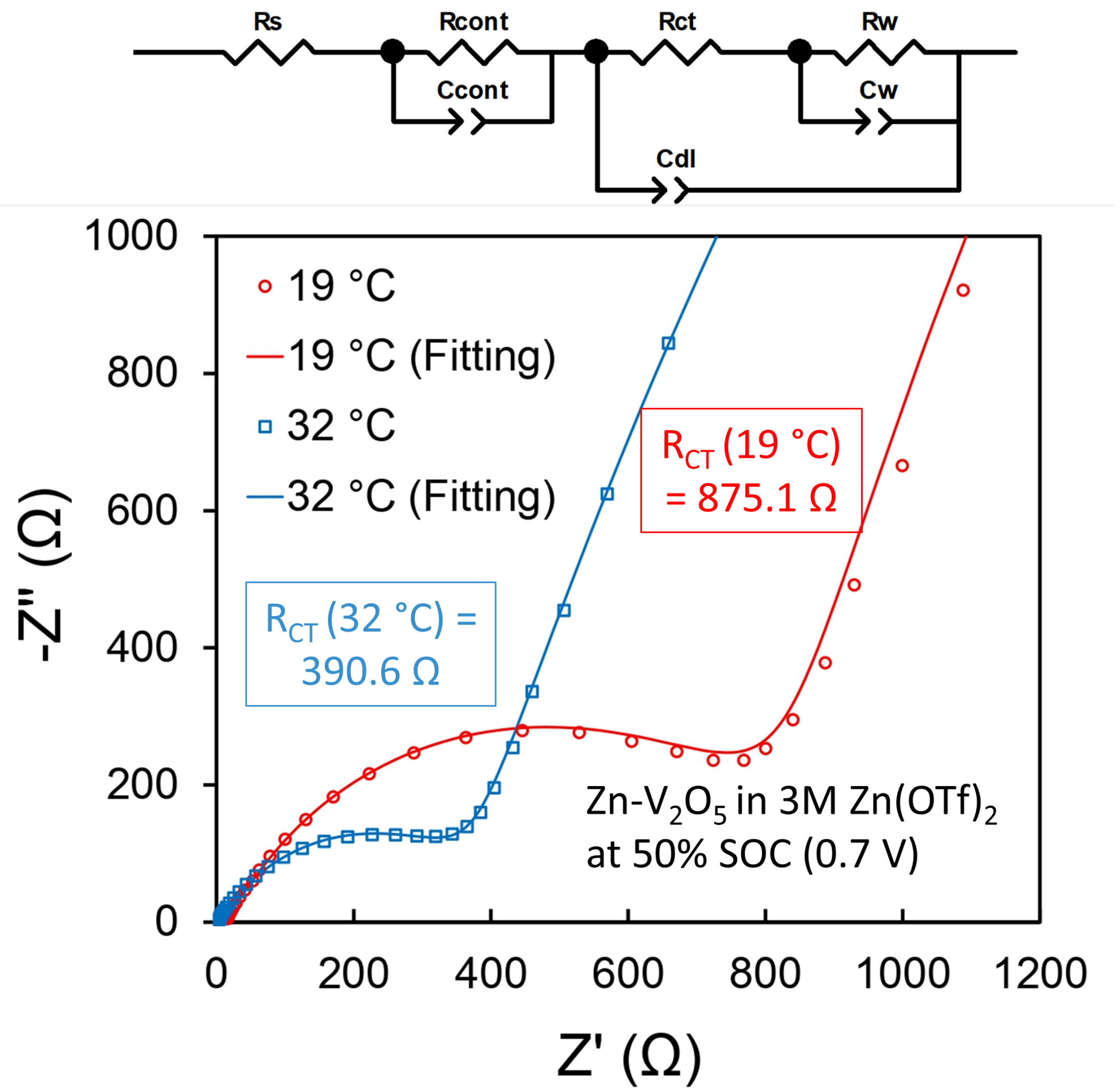

Figure S1: Nyquist plot showing the charge transfer resistance (R_{CT}) of a Zn-V_2O_5 cell which has been discharge to 0.7 V (50% SOC) as a function of temperature. When the temperature is increased from 19 ^{o}C to 32 ^{o}C to mimic the thermal effects of 1 sun irradiation, the charge transfer resistance decreases from 875.1 Ω to 390.6 Ω highlighting that temperature can reduce impedance and improve cell cycling performance. The equivalent circuit used for EIS fitting is shown on the top of the figure.

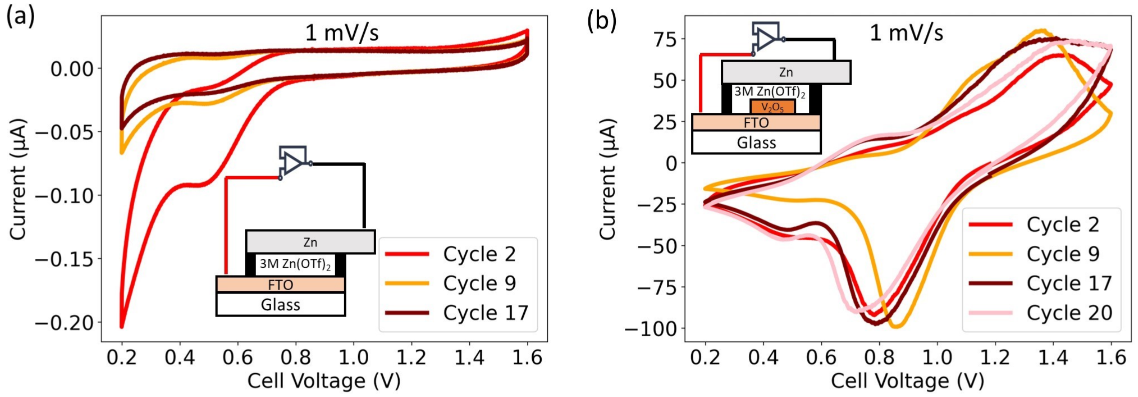

Figure S2: (a) CV curves for FTO glass cycled against a zinc metal anode in 3M $Zn(OTf)_2$ electrolyte at a scan rate of $1 mVs^{-1}$ in a voltage range between 0.2-1.6 V. Side reactions are seen at a voltage between 0.2-0.6 V for the initial cycles, however, the intensity of these peaks decreases with an increasing number of cycles. (b) CV curves for a V_2O_5 cathode dropcasted on FTO glass cycled against a zinc metal anode in the same electrolyte at the same scan rate. The overpotentials of the system continuously increase with the number of cycles as evidenced by the widening peak-to-peak shift indicating the instability of the system.

Supplementary Note 5 - Choosing the right current collector for zinc ion photobatteries Although DSSCs and some photobattery publications use fluorine-doped tin oxide (FTO) as a current collector due to its transparent and conductive nature, we found that cycling the cell between 0.2-1.6 V against a zinc metal anode in a 3M $Zn(OTf)_2$ electrolyte leads to side reactions at a voltage between 0.2-0.6 V (Figure S2(a)). This leads to a degradation in cell performance as evidenced by increased overpotentials and peak-to-peak width (Figure S2(b)). Hence, FTO is not a stable substrate in this potential range.

Instead, we use a thick carbon layer over the dropcast cathode as a current collector. Despite the thick layer, the deposited carbon is porous allowing for electrolyte penetration, and has a high in-plane electrical conductivity (sheet resistance $< 15\Omega/\square$ for a 25µm thick film). The planar cell structure shows good cycling performance over 80 cycles (Figure S4) and does not show any of the degradation problems experienced by FTO. Additionally, the flat structure and large area of the cells allow for easier *operando* optical spectroscopy and microscopy measurements.

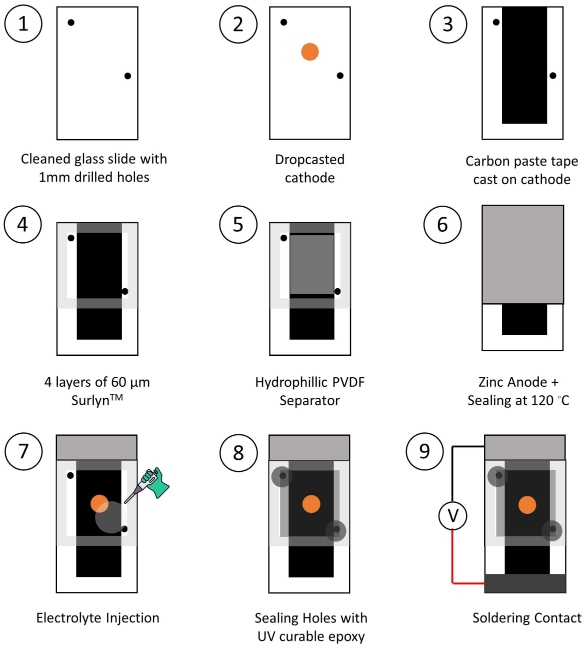

Figure S3: The steps involved in the fabrication of planar cells. First, 1 mm holes are drilled on a pre-cut glass slide. The glass is the washed in acetone, IPA, and DI water. The cathode slurry is dropped on the glass slide followed by drying on a hotplate. Then, carbon paste is tape caste on the cathode to act as a current collector and dried on a hotplate. 4 layers of thermoplastic sealant are placed on the carbon layer followed by a PVDF membrane as a separator. The zinc anode is placed on the thermoplastic layers and the entire structure is sealed on a hotplate. The electrolyte is injected through one of the drilled holes until the entire space is filled. Finally, the holes are sealed with UV curable epoxy and an ultrasonic welder is used to deposit a metallic contact with the carbon paste.

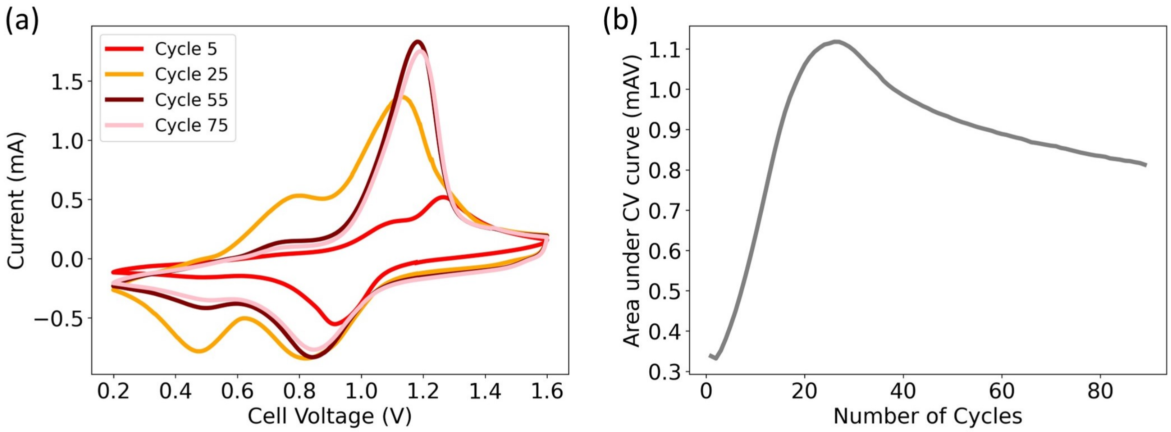

Figure S4: (a) Cyclic voltammetry scans (80 cycles) at a scan rate of 1 mVs^{-1} for a V_2O_5-Zn planar cell and (b) The area under the CV curves as a function of the number of cycles. The battery displays stable operation with a characteristic 'hump' associated with the ageing of V_2O_5 and does not show a continued increase in overpotential as seen when FTO is used as a transparent conductive substrate.

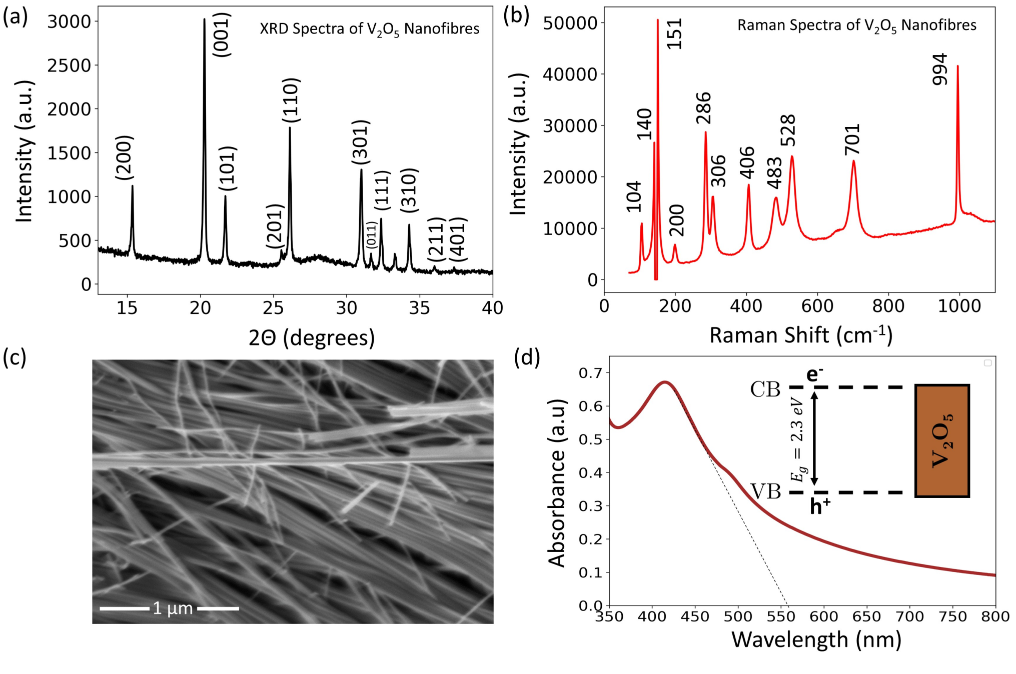

Figure S5: (a) XRD spectrum of the V_2O_5 nanowires synthesized hydrothermally (b) Raman spectra of the V_2O_5 nanowires (c) SEM images of showing the nanowire like morphology of the V_2O_5 (d) UV-vis spectra of the nanowires revealing a band gap of about 2.3 eV.

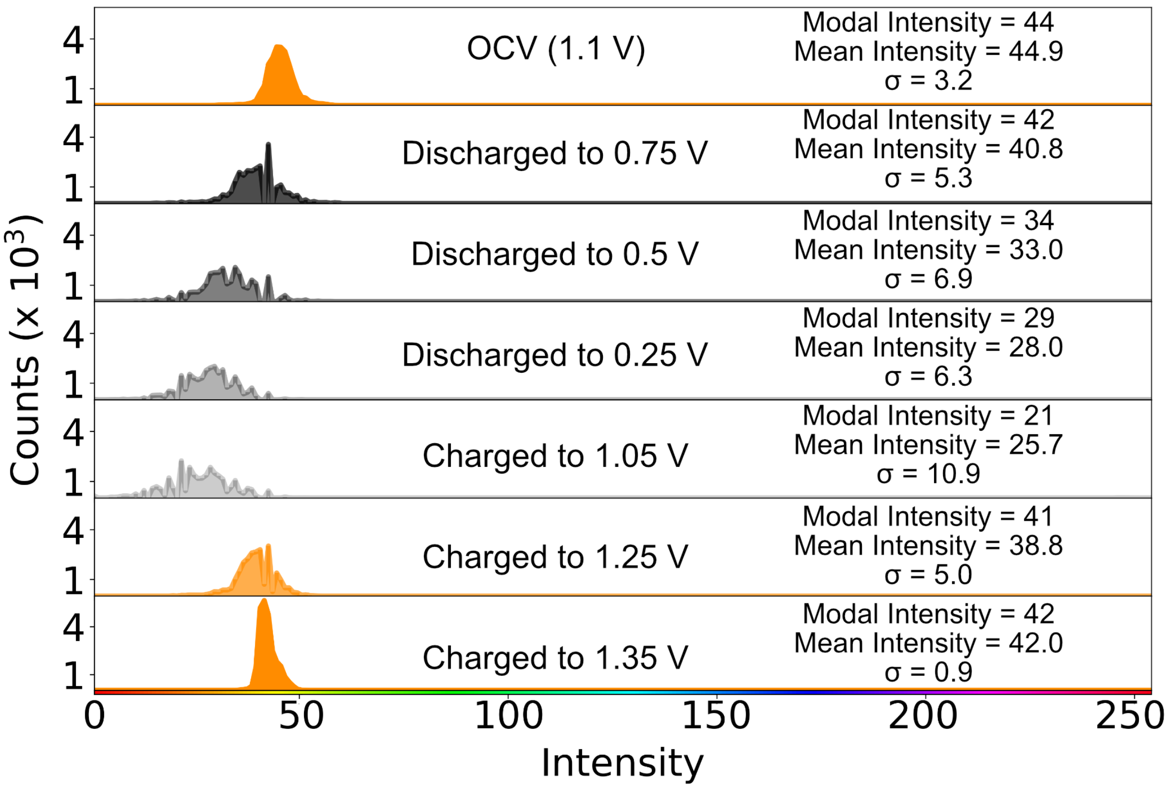

Figure S6: Histograms of hue for a 100x100 micron subsection of the optical microscopy images plotted at different states of charge. A 4-sigma Gaussian filter was applied before HSV conversion and data extraction. The x-axis corresponds to the hue of the image and the y-axis to the relative contribution to color from each hue. Initially, a narrow distribution of hue intensity is seen with both mean (44.9) and mode (44) corresponding to a yellow hue which matches the visibly observed color. As the cell is discharged, the peak of the histogram shifts to the left and the distribution becomes broader (as evidenced by the increasing standard deviation) which is in accordance with the greyer regions seen. When the cell is charged, the peak of the histogram returns to its initial mode indicating that the colour change is reversible. The difference in intensities in the OCV and charged state is due to slight self-discharge in the cell after assembly and overexposure of the sample towards the end of cycling.

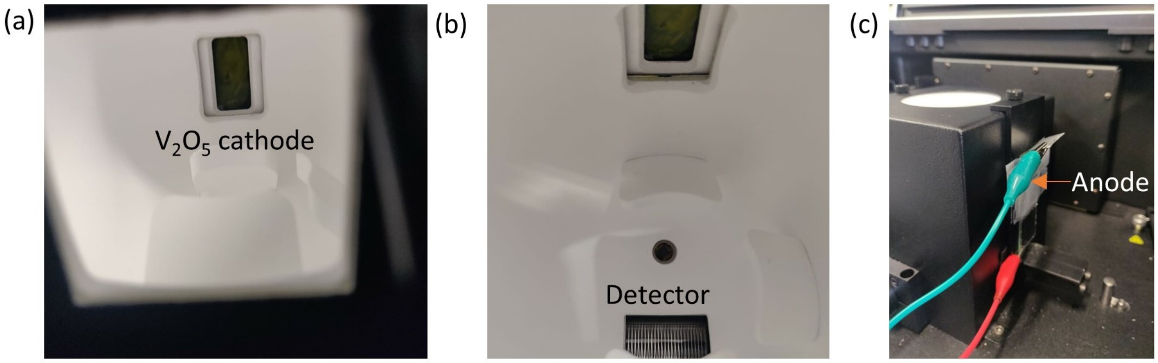

Figure S7: Digital images of (a) the V_2O_5 cathode mounted on the integrating sphere (b) the position of the detector (c) the external connections to the potentiostat - the zinc foil can directly be connected to the negative terminal while an ultrasonic welder is used to provide a metallic connection to the carbon current collector on the cathode.

Supplementary Note 6 - Reflection vs absorption spectroscopy for monitoring optical changes in photocathodes: Although absorption spectroscopy is the most commonly used method to track the band gap of materials, most batteries contain an opaque separator and anode which prevents measurements in absorption mode. Usually, to circumvent this several cells are charged to specific voltages and disassembled. Then, their cathode materials are extracted from the current collectors and washed before their UV-vis spectra are measured. This process is tedious, involves the fabrication of several cells and does not provide real-time information about band gaps or the effect of continued cycling on them. In reflection spectroscopy, light reflected from the cathode is measured using a detector, allowing for non-invasive measurements of the optical properties of the cathode. Reflection data can be interpreted as the complement of absorption data - a peak in reflectance is associated with an absorption onset due to the associated modulation of the refractive index of the material. Hence, regions showing a dip in reflectance imply increased absorption and sharp increases in reflectance occurs at band edges.

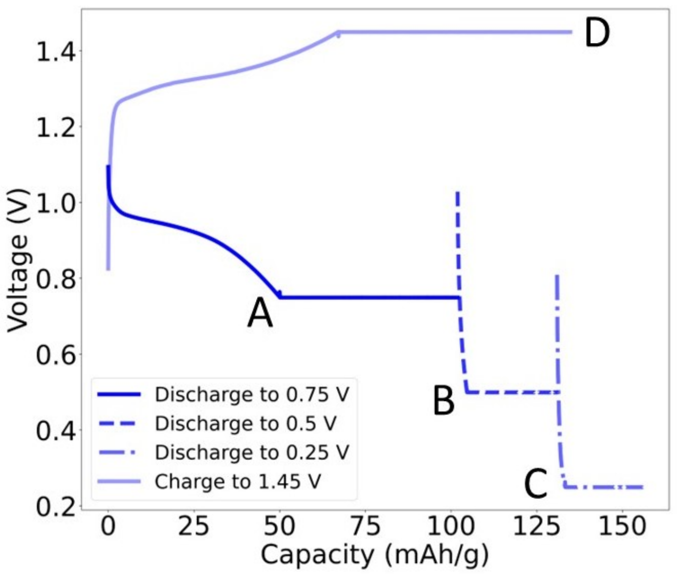

Figure S8: The discharge/charge protocol used for operando reflection spectroscopy. The cell was discharged at a current density of 80mAg^{-1} to 0.75 V followed by a 2 hour voltage hold at 0.75 V (Point A), then to 0.5 V followed by a 2 hour voltage hold at 0.5 V (Point B), then to 0.25 V followed by a 2 hour voltage hold at 0.25 V (Point C) and finally charged at the same current density to 1.4 V followed by a 2 hour voltage hold (Point D). Reflection measurements of the photocathode are carried out at each of these points. The cell shows high overpotentials due to the low proportion of conductive additives in the electrode slurry (1%).

Supplementary Note 7 - Degenerate doping of photocathodes: When the cell is discharged to 0.75 V, the discharge capacity of the cell is 100 $\mathrm{m\,A\,h\,g^{-1}}$ which corresponds to 6.1x10^{21} e^- cm^{-3} of inserted charge (assuming the density of V_2O_5 is 3.36 g cm^{-3}). It should be noted here that some of the inserted electrons will be used for side reactions such as solid electrolyte interface (SEI) formation. The degenerate doping limit (the point at which large amounts of doping result in semiconductors transitioning to metals) of most semiconductors is of the order of 10^{18} e^- cm^{-3} [5] which means that the limit of degenerate doping of the semiconductor has been surpassed

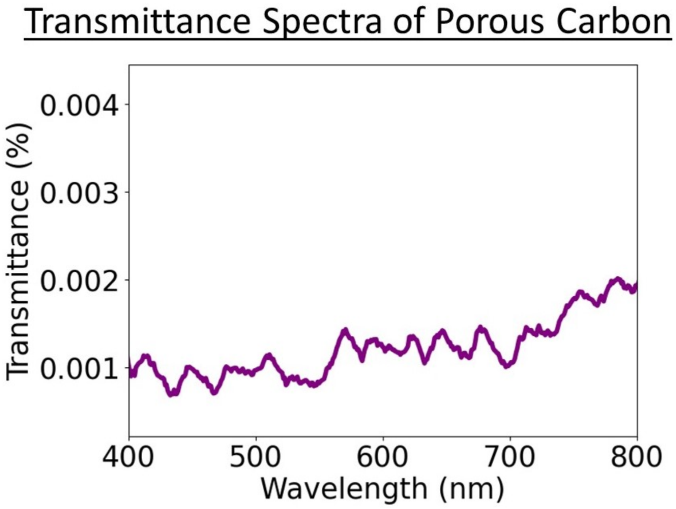

Figure S9: The transmittance of porous carbon - the transmittance is almost zero across the wavelength range tested, indicating that no light can pass through.

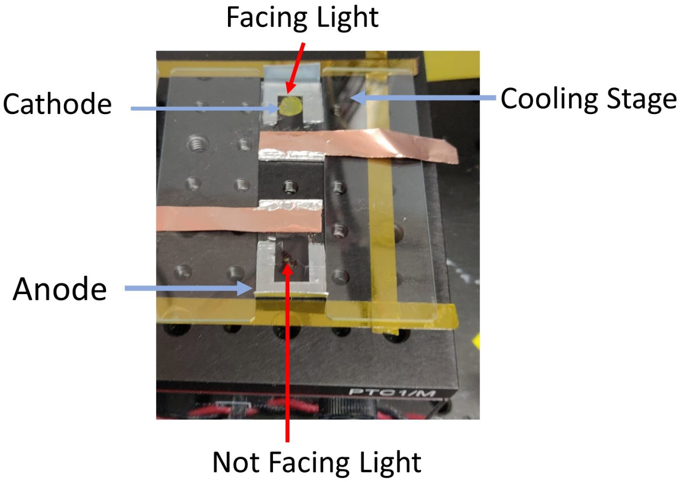

Figure S10: The layout of the cells used for control experiments. The metallic zinc anode was placed on the cooling stage. Two types of cells were fabricated, one where the active material was facing light and one where the active material was covered by a thick layer of porous carbon. As the transmittance of the carbon layer is negligible, any photo-enhanced effects seen must be due to thermal effects.

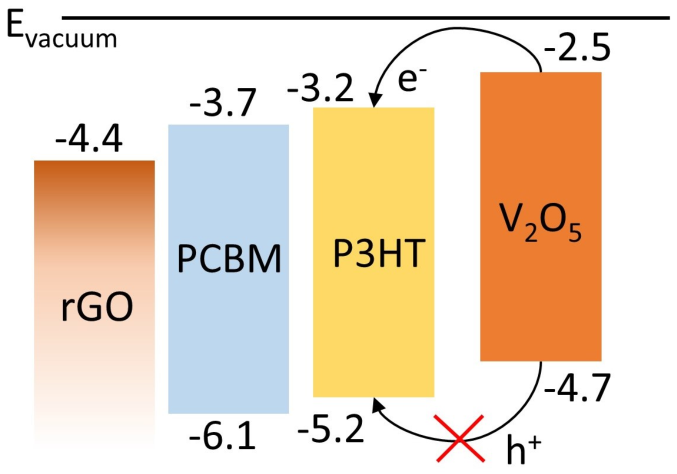

Figure S11: The band diagram of the photocathode slurry. The band alignment between P3HT and V_2O_5 enables photoelectrons to be effectively extracted from the conduction band while holes are blocked.

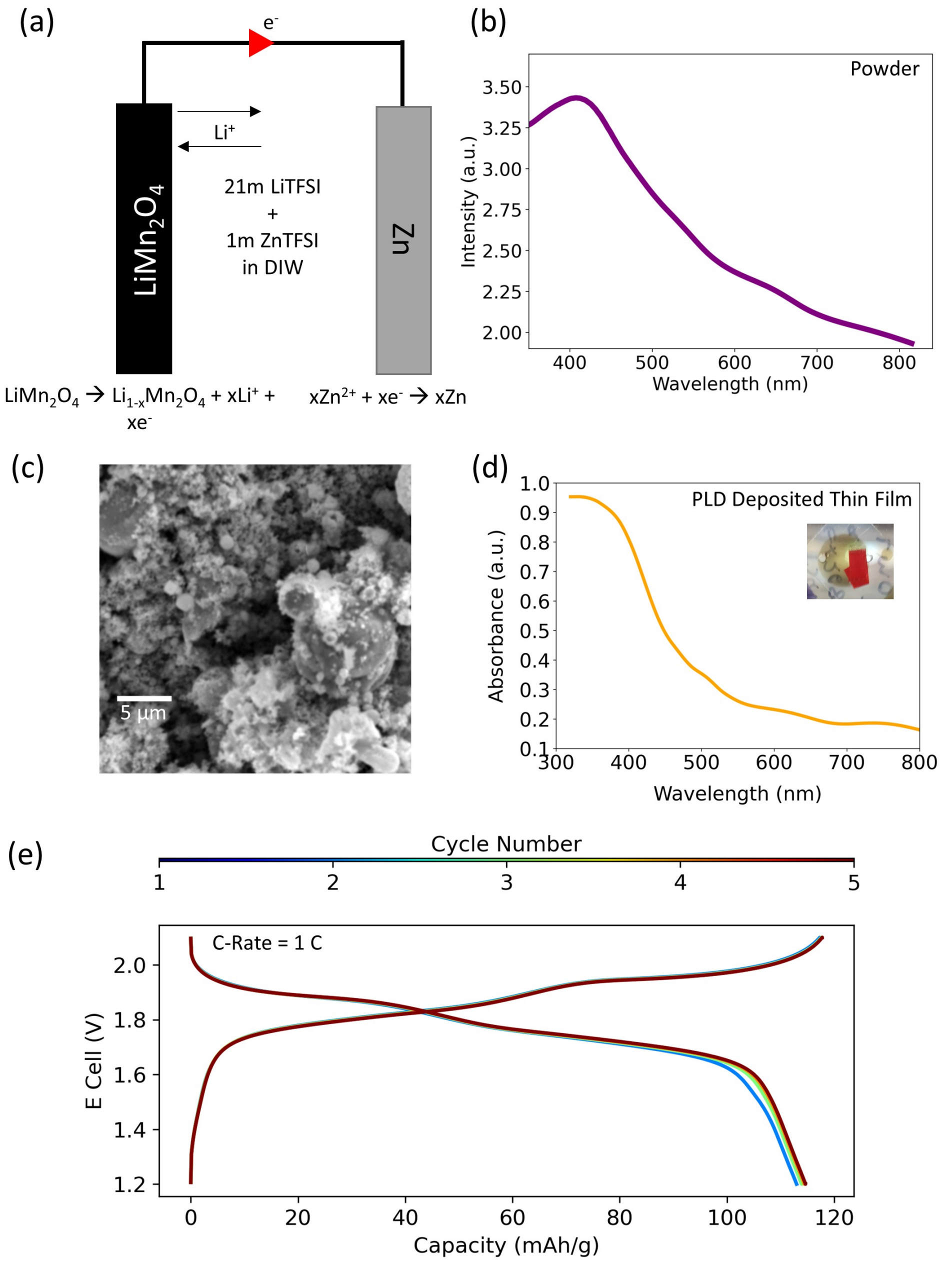

Figure S12: (a) The electrochemistry of $LiMn_2O_4$-Zn batteries. A $LiMn_2O_4$ cathode is used against a Zn anode in a water in salt electrolyte consisting of both lithium and zinc salts. During the charging process, lithium ions are de-inserted from the cathode, and zinc ions are plated on the anode for charge compensation. (b) The absorption spectra of $LiMn_2O_4$ showing a band gap of about 2 eV. (c) SEM images of the $LiMn_2O_4$ particles displaying an average particle size between 300-1000 nm. (d) The absorption spectra of PLD deposited $LiMn_2O_4$ thin films. (e) Cycling performance of planar $LiMn_2O_4$-Zn cells, showing stable performance over 5 cycles at 1C.

Supplementary Note 8 - The $LiMn_2O_4$ -Zn planar cell: Here, we use a highly concentrated water-in-salt electrolyte (21 mol.g^{-1} LiTFSI + 1 mol.g^{-1} $Zn(TFSI)_2$ in DI

water) which enables the insertion of lithium ions in and out of the cathode accompanied by the simultaneous stripping and plating of zinc ions at the anode. A schematic of this electrochemical system is shown Figure S12(a). We used commercial $LiMn_2O_4$ nanopowder which displayed a band gap of about 2 eV (Figure S12(b)). However, due to the particle size of the $LiMn_2O_4$ (300-1000 nm) as seen through SEM (Figure S12(c)) we expect strong scattering effects to be present in the absorption spectra. Therefore, we also provide the absorption spectrum of a PLD deposited $LiMn_2O_4$ thin film which displays a band gap of about 2.6 eV (Figure S12(d)). Figure S12(e) shows the cycling performance of $LiMn_2O_4$-Zn planar cells for 5 cycles at 1C indicating stable electrochemical performance and a consistent discharge capacity.

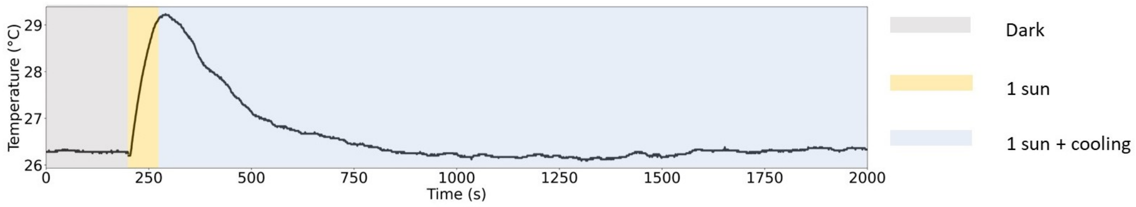

Figure S13: The temperature profile of the planar cell used for temperature controls. Initially, a steady state temperature of about 26.1 ^{O}C is observed. After the solar simulator is switched on, the temperature of the cell rises rapidly. To counter this temperature rise, the cooling stage is set to a temperature of 16 ^{O}C. This results in the rate of temperature rise decreasing and the temperature eventually reaching it's steady state value of 26.1 ^{O}C allowing for measurements of photoenhanced capacities while subtracting thermal contributions.

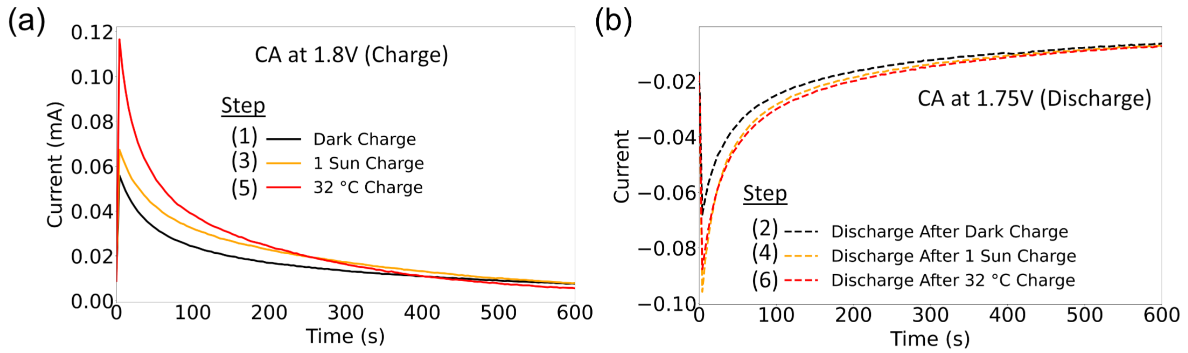

Figure S14: An LMO-Zn cell was charged to 1.75 V by applying a constant current followed by a voltage hold at 1.75 V. (a) Chronoamperometry (CA) curves of the cell when charged by applying a voltage of 1.8 V under dark (Step 1), light (Step 3) and heated (Step 5) conditions. (b) Dark discharge of the cell by applying a voltage of 1.75 V after dark (Step 2), light (Step 4), and heated (Step 6) charge. An increase in charge and discharge capacity is seen for both the illuminated and heated cells, similar to that seen under constant current.

Supplementary Note 9 - Chronoamperometry (CA) measurements under light, dark, and heated conditions: The CA measurements were performed using voltage holds at 1.8 V. The peak currents observed under 1 sun and heated conditions increase when compared to the dark, indicating that both heat and light can aid lithium-ion diffusion. When discharged by applying a voltage of 1.75 V, both the cells that were heated and illuminated show similar discharge curves, with a higher discharge current than the cell charged in the dark. This indicates again that both light and heat can increase the capacity of the cell.

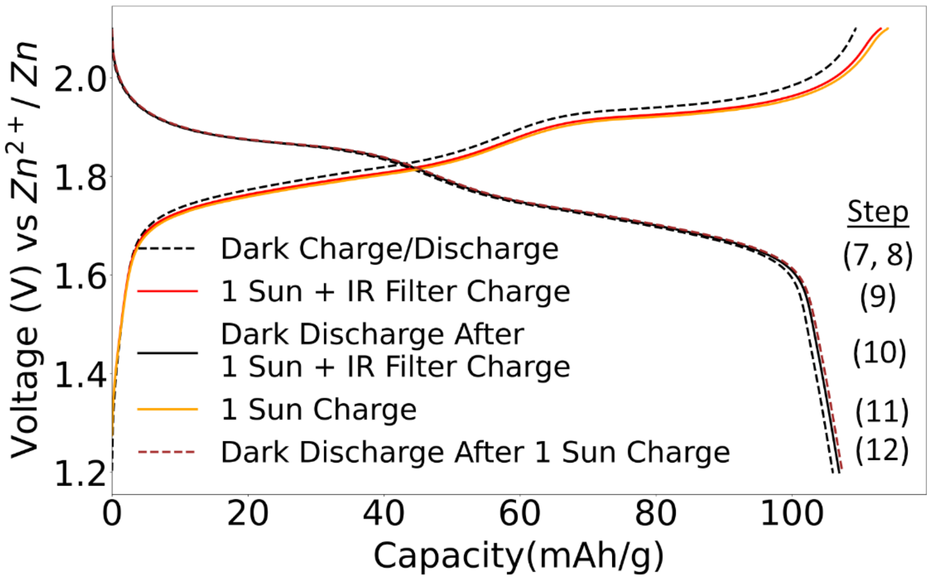

Figure S15: Constant current charge and discharge on an LMO-Zn cell under dark conditions, 1 sun, and 1 sun with an IR filter (all wavelengths above 700 nm blocked out). A summary of capacities is provided in Table S3. The discharge capacity obtained after using the IR filter is higher than the cell in the dark but lower than the cell charged under 1 sun. Therefore, IR heating is responsible for some but not all of the capacity enhancements seen.

Supplementary Note 10 - The role of infrared radiation in photoelectrochemical measurements: We assess the contribution of infrared (IR) light to the enhancement in electrochemical performance under 1 sun conditions as infrared light cannot create electron hole pairs in the material but can contribute to heating. We use 700 nm and 800 nm shortpass filters to prevent IR light from reaching our cells. Our results are presented in Table S3 and Figure S15. A discharge capacity of 106.0 $\mathrm{m\,A\,h\,g^{-1}}$ in the dark, 106.9 $\mathrm{m\,A\,h\,g^{-1}}$ under 1 sun without IR light and 107.4 $\mathrm{m\,A\,h\,g^{-1}}$ under 1 sun illumination is obtained. Although cutting off IR light reduces the photoenhancement seen, the capacity obtained under IR light is still higher than that seen in the dark. Additionally, the temperature of the Zn anode under these conditions is measured to be 31 ^{o}C (the room temperature was 24 ^{o}C). Therefore, we conclude that visible light can also contribute to heating of the cell, either due to absorptions by broadband absorbers such as carbon black present in the electrode or thermalization of above-band gap energy photons through the aforementioned photothermal effect. It

appears that merely cutting off IR light is insufficient to prevent heating of the cell.

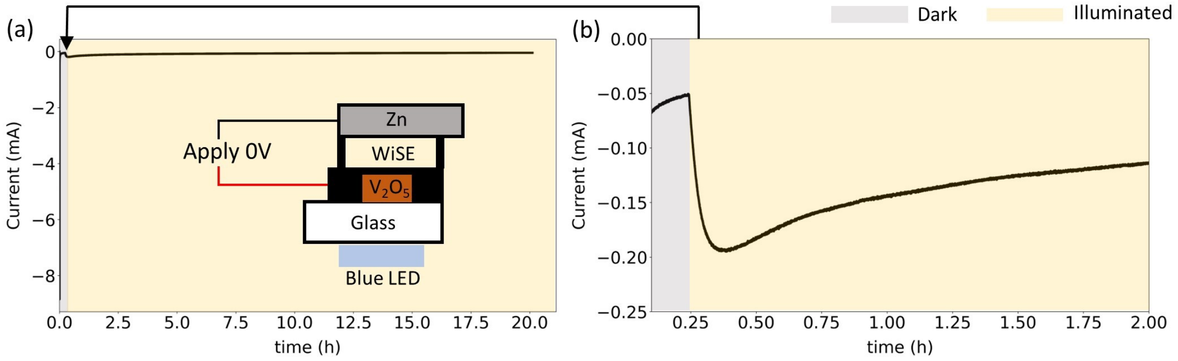

Figure S16: (a) Current vs time graphs for a V_2O_5-Zn battery being photocharged. After discharging the cell to 0 V, a potential of 0 V is applied between the anode and the cathode to short circuit the cell. Initially, a large negative current is seen. Then, a blue LED is used to illuminate the cell. (b) a zoomed in picture of (a). After the light is switched on, the negative current recorded increases, indicating that the light is contributing to a higher rate of discharge of the cell, rather than photocharging the cell.

Supplementary Note 11 - Photocharging the V_2O_5-Zn system Here, we check whether the V_2O_5-Zn system can be photocharged - i.e. whether the battery can be charged using only light an no external bias under short-circuit conditions. To test this, a V_2O_5 photocathode with a was assembled against a Zn anode. P3HT and PCBM can act as electron transport layers according to the energy diagram shown in Figure S11. The cell was discharged to 0 V and then the cell was short-circuited by applying a potential of 0 V between the anode and the cathode using a potentiostat to allow for a path for photogenerated electrons to flow from the cathode to the anode (Figure S16). When the cell is illuminated with a blue LED (intensity $\approx$ 100 mW cm^{-2}, λ=455 nm) the negative current increases, indicating that the cell is not being photocharged and that the light is contributing to a higher discharge current for the cell, probably due to lowered resistance of the cathode upon illumination. Therefore, the V_2O_5-Zn system cannot be directly photocharged and only photoassisted charge/discharge measurements can be performed on it.

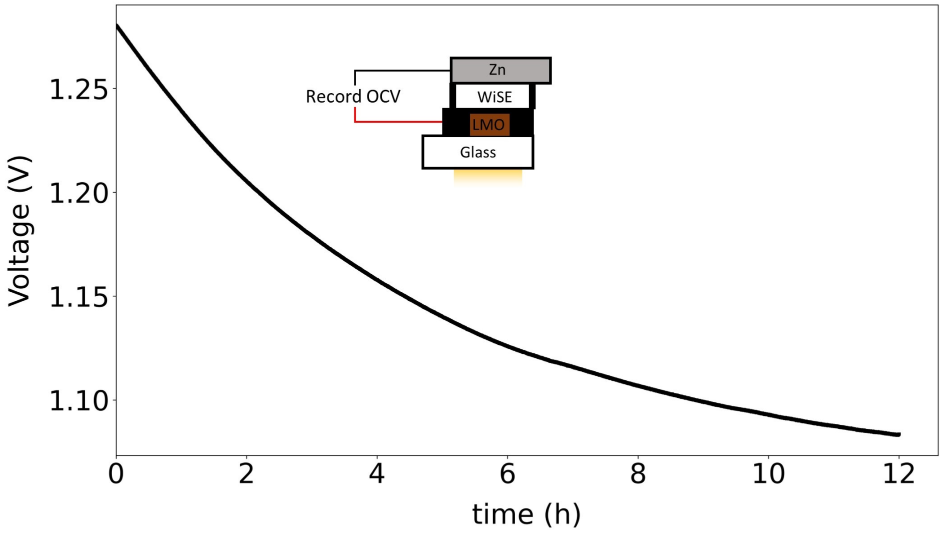

Figure S17: The change in open circuit voltage of an $LiMn_2O_4$-Zn illuminated after being discharged. A continuous decrease in OCV is seen for the illumination period.

References

[1] L. Zhang, C. Jiang, C. Wu, H. Ju, G. Jiang, W. Liu, C. Zhu, and T. Chen, "V_2O_5 as hole transporting material for efficient all inorganic Sb_2S_3 solar cells," *ACS Applied Materials & Interfaces*, vol. 10, pp. 27098–27105, 2018.

[2] T. Trupke, M. Green, and P. Würfel, "Improving solar cell efficiencies by down-conversion of high-energy photons," *Journal of Applied Physics*, vol. 92, pp. 1668–1674, 2002.

[3] W. Gärtner, "Photothermal effect in semiconductors," *Physical Review*, vol. 122, p. 419, 1961.

[4] N. Zhang, Y. Dong, M. Jia, X. Bian, Y. Wang, M. Qiu, J. Xu, Y. Liu, L. Jiao, and F. Cheng, "Rechargeable aqueous Zn–V_2O_5 battery with high energy density and long cycle life," *ACS Energy Letters*, vol. 3, pp. 1366–1372, 2018.

[5] C. Kittel, *Introduction to solid state physics*. John Wiley & Sons, inc, 2005.
